# Fast demographic traits promote high diversification rates of Amazonian trees

**DOI:** 10.1111/ele.12252

**Published:** 2014-03-03

**Authors:** Timothy R Baker, R Toby Pennington, Susana Magallon, Emanuel Gloor, William F Laurance, Miguel Alexiades, Esteban Alvarez, Alejandro Araujo, Eric J M M Arets, Gerardo Aymard, Atila Alves de Oliveira, Iêda Amaral, Luzmila Arroyo, Damien Bonal, Roel J W Brienen, Jerome Chave, Kyle G Dexter, Anthony Di Fiore, Eduardo Eler, Ted R Feldpausch, Leandro Ferreira, Gabriela Lopez-Gonzalez, Geertje van der Heijden, Niro Higuchi, Eurídice Honorio, Isau Huamantupa, Tim J Killeen, Susan Laurance, Claudio Leaño, Simon L Lewis, Yadvinder Malhi, Beatriz Schwantes Marimon, Ben Hur Marimon Junior, Abel Monteagudo Mendoza, David Neill, Maria Cristina Peñuela-Mora, Nigel Pitman, Adriana Prieto, Carlos A Quesada, Fredy Ramírez, Hirma Ramírez Angulo, Agustin Rudas, Ademir R Ruschel, Rafael P Salomão, Ana Segalin de Andrade, J Natalino M Silva, Marcos Silveira, Marcelo F Simon, Wilson Spironello, Hans ter Steege, John Terborgh, Marisol Toledo, Armando Torres-Lezama, Rodolfo Vasquez, Ima Célia Guimarães Vieira, Emilio Vilanova, Vincent A Vos, Oliver L Phillips, John Wiens

**Affiliations:** 1 School of Geography, University of LeedsLeeds, LS2 9JT, UK; 2 Royal Botanic Garden EdinburghEdinburgh, EH3 5LR, UK; 3 Instituto de Biología, Universidad Nacional Autónoma de MéxicoMexico City, Mexico; 4 Centre for Tropical Environmental and Sustainability Science (TESS) and School of Marine and Tropical Biology, James Cook UniversityCairns, Queensland 4878, Australia; 5 School of Anthropology and Conservation, University of KentCanterbury, Kent CT2 7NR, UK; 6 Facultad de Ingenieria Forestal, Universidad del TolimaIbaqué, Colombia; 7 Museo de Historia Natural Noel Kempff MercadoSanta Cruz, Bolivia; 8 Alterra, Wageningen University and Research Centre, 6708 PBWageningen, The Netherlands; 9 Herbario Universitario PORTGuanare, Venezuela; 10 Projeto TEAM – Manaus, Instituto Nacional de Pesquisas da AmazôniaCEP 69067-375, Manaus, Brazil; 11 INRA, UMR EEF INRA-Université de Lorraine54280, Champenoux, France; 12 CNRS and Université Paul Sabatier, UMR 5174 EDB31062, Toulouse, France; 13 School of GeoSciences, University of EdinburghEdinburgh, EH9 3JW, UK; 14 Department of Anthropology, University of Texas at AustinAustin TX 78712, USA; 15 Museu Paraense Emílio GoeldiCEP 66040-170, Belém, Brazil; 16 School of Freshwater Sciences, University of Wisconsin-MilwaukeePO Box 413, WI 53201, USA; 17 Smithsonian Tropical Research InstituteApartado Postal 0843-03092 Panama, Republic of Panama; 18 Instituto Nacional de Pesquisas da AmazôniaCEP 69067-375, Manaus, Brazil; 19 Instituto de Investigaciónes de la Amazonía PeruanaIquitos, Perú; 20 Herbario CUZ, Universidad Nacional San Antonio Abad del CuscoPerú; 21 Conservation InternationalArlington, Virginia, VA 22202, USA; 22 Instituto Boliviano de Investigación ForestalSanta Cruz, Bolivia; 23 Department of Geography, University College LondonLondon, WC1E 6BT, UK; 24 Environmental Change Institute, School of Geography and the EnvironmentUniversity of Oxford, Oxford OX1 3QY, UK; 25 Universidade do Estado de Mato Grosso - Campus de Nova Xavantina, CEP 78690-000, Nova XavantinaBrazil; 26 Jardín Botanico de MissouriOxapampa, Peru; 27 Universidad Estatal AmazónicaPuyo, Ecuador; 28 Universidad Nacional de ColombiaSede Amazonia, Leticia, Colombia; 29 Center for Tropical Conservation, Nicholas School of the Environment, Duke UniversityDurham NC 27705, USA; 30 Instituto de Ciencias Naturales, UNALBogota, Colombia; 31 Universidad Nacional de la Amazonía PeruanaIquitos, Peru; 32 INDEFOR, Universidad de los AndesMérida, Venezuela; 33 Embrapa Amazônia OrientalCEP 660-95100, Belém, Brazil; 34 PDBFF, Instituto Nacional de Pesquisas da AmazôniaCEP 69067-375, Manaus, Brazil; 35 Universidade Federal Rural da AmazôniaCEP 66077-830, Belém, Brazil; 36 Instituto Floresta TropicalCEP 66025-660, Belém, Brazil; 37 Museu Universitário, Universidade Federal do AcreCEP 69920-900, Rio Branco, Brazil; 38 Embrapa Recursos Genéticos e BiotecnologiaCEP 70770-900, Brasília, Brazil; 39 Naturalis Biodiversity Center2333 CR Leiden, The Netherlands; 40 Department of Biology, Ecology and Biodiversity Group, Utrecht University3508 TB Utrecht, The Netherlands; 41 Universidad Autónoma del BeniRiberalta, Bolivia

**Keywords:** Diversity, generation time, traits, tropical forest, turnover

## Abstract

The Amazon rain forest sustains the world's highest tree diversity, but it remains unclear why some clades of trees are hyperdiverse, whereas others are not. Using dated phylogenies, estimates of current species richness and trait and demographic data from a large network of forest plots, we show that fast demographic traits – short turnover times – are associated with high diversification rates across 51 clades of canopy trees. This relationship is robust to assuming that diversification rates are either constant or decline over time, and occurs in a wide range of Neotropical tree lineages. This finding reveals the crucial role of intrinsic, ecological variation among clades for understanding the origin of the remarkable diversity of Amazonian trees and forests.

## Introduction

Amazonian forests are among the most biologically diverse ecosystems on Earth, sustaining approximately 16 000 species of trees or ≈ 30% of global tree diversity (Fine *et al.* 2009; ter Steege *et al.* 2013) with some communities containing over 300 species of at least 10 cm diameter at breast height (dbh) within a single hectare (Gentry 1988). This diversity is a result of an interaction between extrinsic factors – historical events that have caused extinction or provided opportunities for speciation – and the intrinsic characteristics of different lineages that have influenced how they respond to these events (Vamosi & Vamosi 2011). For Amazonia, there has been a strong focus on identifying the role that extrinsic factors have played in promoting high speciation rates associated with the uplift of the Andes, climatic variation, or the development of diverse edaphic conditions (Hoorn *et al.* 2010). Indeed, a wide range of these factors is likely to have been important across different groups: recent phylogenetic studies have shown that speciation events related to historical events throughout the Cenozoic have generated the high tree diversity observed today (Hoorn *et al.* 2010). However, a framework based solely on extrinsic factors cannot explain some of the most noteworthy aspects of Amazonian tree biodiversity: the wide variation in rates of diversification among clades (e.g. Couvreur *et al.* 2010) and the existence of a number of species-rich groups with high diversification rates in unrelated lineages (e.g. *Inga* ≈ 300 species; *Guatteria* ≈ 265 species; Pennington *et al.* 2004). These patterns suggest that the intrinsic characteristics of clades should be considered when trying to understand why some clades are so species rich (Marzluff & Dial 1991).

The search for intrinsic, ecological traits to explain variation in species richness among clades has a long history and a range of morphological and life-history traits have been shown to correlate with patterns of species richness and diversification rates among plants (Givnish 2010). For example, poorly dispersed seeds, larger geographic range sizes, monoecious breeding systems and fast demographic traits such as short generation times are all related to higher diversification rates within clades, presumably because these factors increase the probability of reproductive isolation (Givnish 2010; Vamosi & Vamosi 2011). However, there are only a few studies of the ecological correlates of diversification in trees (Marzluff & Dial 1991; Verdu 2002) and none that have focussed on species-rich tropical forests. The lack of studies for tropical trees reflects the paucity of data concerning the life-history strategies and evolutionary relationships within these groups until the recent emergence of large demographic and trait databases (e.g. Lopez-Gonzalez *et al.* 2009) and dated phylogenies for a range of clades (e.g. Erkens *et al.* 2007; Simon *et al.* 2009; Couvreur *et al.* 2010).

Testing the relationships between ecological factors and the diversification rate (*r*), the difference between the rate of speciation (*λ*) and extinction (μ), requires an underlying model of how these processes vary over time. In many studies, diversification has been calculated based on a constant rate, birth/death process that assumes that, on average, the number of species within a clade increases exponentially over time (Magallon & Sanderson 2001). However, based on observations that the rate of appearance of new taxa in the fossil record often declines over time and that at certain scales, clade age and species richness are not always correlated across extant lineages (Rabosky 2009b; Rabosky *et al.* 2012), it has been suggested that ‘density-dependent’ diversification, where the rate of diversification slows down as species accumulate, may be a more appropriate model for some clades. Here, we explicitly test whether ecological factors improve estimates of current species richness using models that are based on either a constant or declining rate of diversification. This approach allows us to determine the model of diversification that is most appropriate for these clades and explore the sensitivity of our results to this assumption.

Understanding the traits associated with high diversification rates may also help explain the origin of current patterns of community-level diversity. In general terms, within any given community the species present may have evolved *in situ*, or arrived via biogeographic dispersal (Moen *et al.* 2009). If the high diversity of western Amazon forests (Gentry 1988) is a result of high levels of *in situ* diversification, we would expect species richness in these forests to be preferentially distributed in clades with traits that are associated with high diversification rates. We test this idea based on our analysis of the correlates of diversification within clades.

## Materials and methods

We searched the literature for dated phylogenies of plant families that contain predominately Neotropical, free-standing, woody genera of dicotyledonous canopy trees to obtain stem or crown node ages for all clades where these data exist. Data were available for 51 clades in eight families that are broadly representative of angiosperm canopy tree diversity in Neotropical forests (Table S1, Doyle *et al.* 2004; Davis *et al.* 2005; Weeks *et al.* 2005; Zerega *et al.* 2005; Muellner *et al.* 2006; Erkens *et al.* 2007; Simon *et al.* 2009; Couvreur *et al.* 2010). Dates were obtained from the authors if not directly available from the publications. Genera known to be polyphyletic were either excluded (*Oxandra*, Annonaceae; *Trophis*, Moraceae; *Stryphnodendron, Acacia*, Fabaceae), or, in cases where two or more polyphyletic genera form clades, the more inclusive, higher level monophyletic groups were used (Neotropical *Protium*, *Crepidospermum* and *Tetragastris*; *Brosimum, Helicostylis* and *Trymatococcus*; *Clarisia* and *Batocarpus*). In addition, genera that include lianas, stranglers or other non-tree growth forms (e.g. *Bauhinia*, *Croton, Ficus*) were excluded. We also compiled estimates of extant species richness for each clade (Pennington *et al.* 2004; The Plant List 2010).

**Figure 1:**
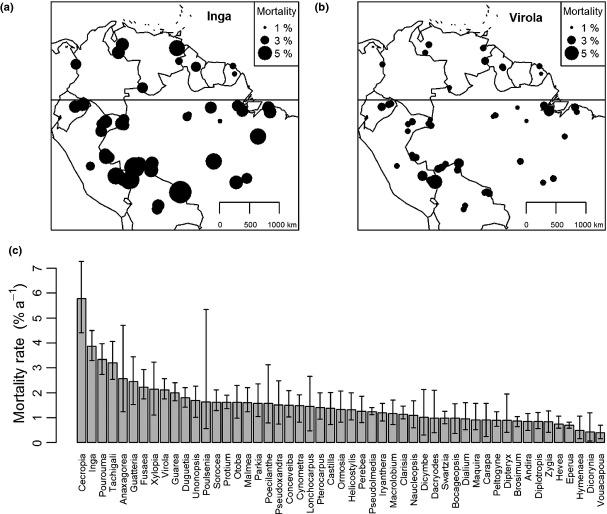
Variation in mortality rates for (a) *Inga* and (b) *Virola* across 57 plot clusters in South American forests; (c) intrinsic mortality rate (± 95% confidence limits) of 51 clades of tropical tree after accounting for variation among plot clusters.

To test whether diversification is related to variation among clades in demographic traits, range size, maximum size, dispersal mode or breeding system, we obtained trait data for each clade. We estimated a measure of the intrinsic demographic rates of each clade – the turnover time of trees ≥ 10 cm diameter (= 1/annual rate of mortality) – using data from 207 long-term, lowland (< 500 m a.m.s.l.), old-growth forest plots (Table S2) that form part of the RAINFOR (Amazon Forest Inventory) network. The plots were established during 1963–2008 and, in total, sample 212.9 ha of forest across the major climatic and edaphic gradients in Amazonia (Table S2). Each plot has been recensused every 4–5 years; the average length of monitoring is 14.6 years (Table S2). The data were extracted from the ForestPlots.net database (Lopez-Gonzalez *et al.* 2009, 2011). Each census comprises diameter measurements of all living trees ≥ 10 cm dbh, and includes records of tree mortality and measurements and identifications of all new recruits. We used data only from plots with annual precipitation > 1300 mm a^−1^ based on the WorldClim dataset (Hijmans *et al.* 2005) and basal area > 13 m^2^ ha^−1^ to exclude plots in dry forest and savanna biomes. All clades contained > 100 individuals, after excluding trees monitored for less than 2 years, which allows reasonable estimates of clade-specific mortality rates (Rüger *et al.* 2011). Due to the typical 1 ha size of forest plots in the RAINFOR network, the impact of large disturbances on landscape-scale tree mortality rates may be underestimated. However, in the context of variation in annual tree mortality rates across Amazonia (0.5–4%, Phillips *et al.* 2004) and among clades (0.5–6%, Fig. 1), the magnitude of this effect is small (e.g. accounting for this effect in forests near Manaus leads to an increase in estimated community-level mortality rates from 1 to 1.2%, Chambers *et al.* 2013).

Tree mortality rates vary both due to the intrinsic, ecological characteristics of the species/clade being studied and extrinsic, environmental conditions. For tropical trees, intrinsic variation among clades primarily reflects differences in life-history strategies related to adaptations to different levels of light demand (Turner 2001). In addition, within Amazonia, tree mortality rates are also influenced by an east-west gradient in soil fertility and physical properties (Quesada *et al.* 2012). Here, we disentangled the role of environmental factors and intrinsic controls on tree mortality rates by using mixed models implemented with the *lmerBayes* function (Condit 2012) in R (R Development Core Team 2012).

The survival probabilities of individual trees were modelled as an exponentially declining function of the monitoring period (Condit *et al.* 2006) and we simultaneously fitted the effect of clade, as a fixed factor, and ‘plot cluster’ (plots that occur in the same area and share the same three letter code in Table S2) as a random factor, to separate the effect of the two major sources of variation in the data (Appendix S1). Using ‘plot cluster’ as the random factor in the analysis allowed us to explicitly account for the effect of the broad environmental gradients on tree mortality rates across Amazonia while simultaneously estimating the intrinsic mortality rate of each clade. The data set included 37 090 trees within 51 clades and 57 plot clusters.

Estimates of intrinsic annual mortality rates encompass the full range of life-history strategies among tropical trees (Fig. 1c), from values exceeding 5% for the clade of pioneer trees, *Cecropia*, to very low values < 0.5% (e.g. *Dicorynia*). Mortality rates also vary among plot clusters (Fig. 1a and b). For example, both *Inga* and *Virola* show higher mortality rates in western Amazonia and in transitional forests in southern Brazilian Amazonia, matching known stand-level patterns (Fig. 1a and b, Quesada *et al.* 2012). However, these spatial patterns do not confound the intrinsic variation among clades, with generally higher mortality rates in *Inga* compared to *Virola* throughout the region (Fig. 1). From these estimates of the intrinsic mortality rate, *m*, we calculated turnover times for each clade as 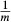.

The range size of each clade was classified as pantropical, Neotropical or Guiana Shield based on Pennington *et al*. (2004). The predominant dispersal type that leads to successful reproduction within each clade was classified as explosive/unassisted, arboreal or ground dwelling mammal, bat or bird, water, or wind, based on the Royal Botanic Gardens Kew Seed Information Database (SID) (2014). The average maximum height per clade, *H*, was calculated from species-level estimates compiled from a range of floras (Baker *et al.* 2009). Breeding system for each clade was classified as dioecious or monoecious based on Pennington *et al*. (2004).

A range of approaches exists for relating traits to variation in diversification, extinction and speciation rates. For large, well-sampled (e.g. > 500 species), well-resolved, species-level phylogenies, likelihood-based approaches can be used to explore associations among traits, the probability of speciation and extinction and the topology of the phylogeny (FitzJohn 2010). Where this level of data are lacking, studies often correlate the average diversification rate under a constant rate model (Magallon & Sanderson 2001) with the traits of interest. Alternatively, correlating log (*N*) with a set of traits, where *N* is the number of extant lineages, has been proposed to explore the controls on diversification among lineages where species richness is not correlated with clade age (Rabosky 2009a).

Although suitable for smaller data sets, neither of these last two methods allow direct tests of how well different underlying models of the diversification process fit the data, or whether the choice of the underlying model affects the significance of any relationships between traits and the diversification rate. Incorporating such tests within these kinds of analyses would help to resolve debates concerning the role of ecological factors in limiting diversification (Rabosky 2009a; Wiens 2011). We therefore compared the role of ecological factors in explaining variation in species richness based on models of both constant and declining rates of diversification building on methods presented in Rabosky (2009b). In contrast to Rabosky (2009b), we compare models based on a range of different traits and estimate the fit of different models within a phylogenetic framework that accounts for the non-independence of different clades. Our approach is similar to Etienne *et al*. (2012) but we focus on developing time-dependent, rather than diversity-dependent, models of diversification.

In general, the mean number of lineages, *N(t)*, from a non-homogeneous diversification process is given as (Bailey 1964, eqn 9.40):(1)
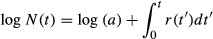
where *a* is the number of ancestral lineages (one for a clade with a stem node age and two for a clade with a crown node age), *t* is the age of the clade (Ma) and *r* is the net diversification rate (events per Ma):(2)
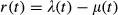
where λ is the speciation rate and μ is the extinction rate.

**Figure 2:**
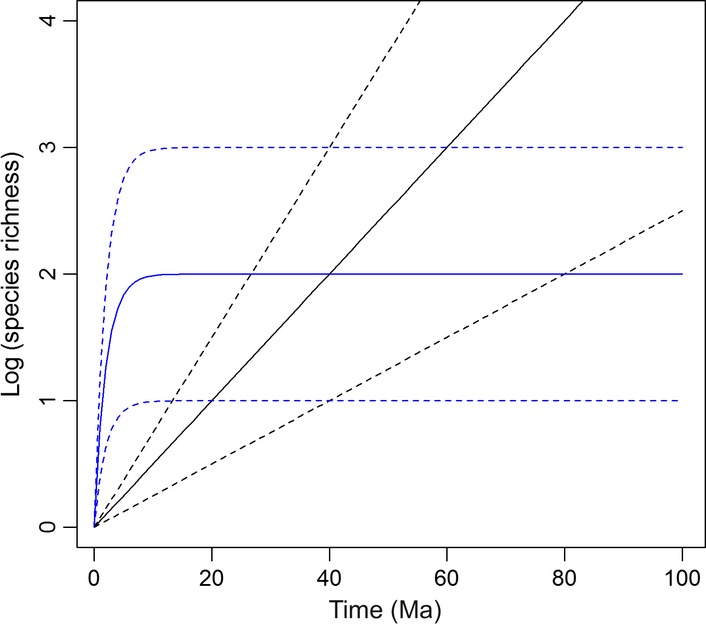
Alternative predictions of the accumulation of species richness by clades under a constant rate (black) and exponentially declining (blue) model of diversification. Each model shows a distinctive relationship between clade age and species richness. Solid lines show the predictions for the null model for each scenario; dashed lines show possible effect of ecological covariates that either promote or reduce diversification (upper/lower lines respectively).

We constructed different estimators of *r*(*t*), based on constant or declining rates of diversification and including and excluding ecological covariates (Fig. 2, Appendix S2). The different estimators of *r*(*t*) were used to predict species richness using eqn (1) and these values were compared with observed values.

We used two forms for the diversification rate (Rabosky 2009b). Firstly, we fit a constant rate model:(3)
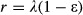
where ε is the relative extinction rate, *μ/λ*, fixed at either a high (ε = 0.9) or low (ε = 0) value.

Secondly, we related the diversification rate to an ecological covariate, *A*:(4)
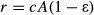


We fit a series of models with *A* represented by either continuous variables, turnover time and maximum height, or factors (range size, dispersal mode and breeding system). We incorporated turnover time and maximum height as 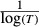 and 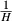, respectively, as we hypothesised that these terms would be negatively correlated with the diversification rate. We used log-transformed values of *T* to ensure that this variable was normally distributed.

Thirdly, we estimated *r* as:(5)
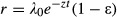
which simulates the diversification rate if speciation and extinction rates decline exponentially over time, consistent with a ‘density-dependent’ model (Fig. 1, Rabosky 2009b). In this model, *λ*_*ο*_ represents the initial diversification rate and *z* is the rate at which diversification slows over time. We also modified this model to allow ecological covariates to influence the initial diversification rate:(6)
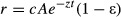
where *A* represents the same ecological covariates as above.

For models based on a continuous variable, we also explored models where the effect of these variables were allowed to vary among the major plant groups (Fabaceae, Moraceae, Annonaceae and other families) represented in our data. We did not fit more complex models involving interactions among terms, or family-level models for categorical variables, as the size of the data set (*n* = 51) precludes effective fitting of more parameters (maximum number of parameters ≈ n/10, Burnham & Anderson 2002).

We fit our models within a phylogenetic framework to account for the non-independence of each clade using phylogenetic generalised least squares regression (Martins & Hansen 1997). Phylogenetic relationships between the groups used in this study (Fig. S1) were obtained from the angiosperm APGIII consensus tree from Phylomatic (Webb & Donoghue 2005) and the branch lengths and relationships modified based on estimated divergence times from TimeTree (Hedges *et al.* 2006). The branch lengths of these phylogenetic relationships were used to estimate the expected correlation matrix among clades based on a Brownian motion model of trait evolution (Appendix S2). We used a transformed version of this matrix, incorporating Pagel's λ (Pagel 1999), to estimate the error structure within our diversification models. This model of trait evolution provided a closer fit to our data than alternative models (Appendix S2). Models were evaluated by comparison of AICc values (Burnham & Anderson 2002). R code to fit these models of diversification is provided in Appendix S2.

We found that one trait – the intrinsic turnover times of different clades – was a useful predictor of diversification rates. To understand the role of variation in this trait for determining community-level patterns of diversity across Amazonia, we explored the contribution that clades with different intrinsic turnover times make to the species richness of western and eastern Amazon forests using the RAINFOR plot data (Table S1).

## Results

**Figure 3:**
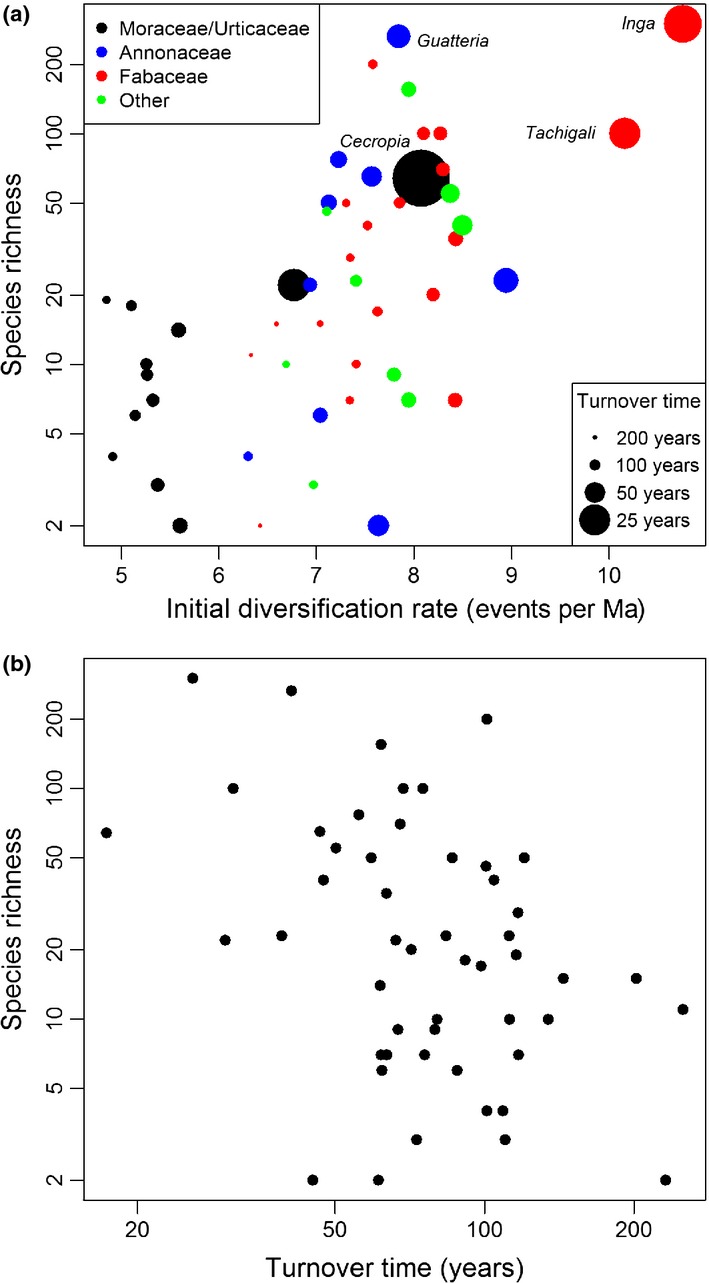
(a) Relationship between observed species richness (natural log scale) and the initial diversification rate of 51 clades of Neotropical trees estimated with the best-fitting model of diversification (Model 15, Table 1, with a low relative extinction rate: ε = 0). For this model, the initial diversification rate is inversely proportional to the intrinsic turnover time of each clade (denoted by symbol size) and declines exponentially over time (*z* estimated as 2.50; eqn 6). (b) Relationship between species richness and intrinsic turnover time of trees ≥ 10 cm dbh across 51 clades (log(species richness) = −0.97*log(turnover time)+7.25, F = 9.11, r^2^  = 0.16).

Turnover time was the only ecological trait that consistently improved predictions of species richness over models of diversification that excluded ecological factors (Table 1): models that linked shorter turnover times with higher diversification rates generated the best estimates of the current species richness of each clade (Table 1, Fig. 3). Of the other ecological factors, only range size provided some improvement to model predictions over our null model (Table 1; using an exponentially declining rate model of diversification), with a small tendency for clades with larger range sizes to have achieved greater species richness. The results were not affected by using scenarios based on either high or low relative extinction rates (Table 1).

**Table 1 tbl1:** AICc values and Pagel's λ for the fit of 16 different models of diversification for 51 clades of tropical trees, with both high (ε = 0.9) and low (ε = 0) relative extinction rates

						ε = 0			ε = 0.9		
Model	Framework	Traits	Family-specific	No. parameters	Lambda	AICc	ΔAICc	r^2^	Lambda	AICc	ΔAICc	r^2^
15	Declining	Turnover time	Yes	7	−0.11	160.58	0.00	33.5	−0.11	163.85	3.27	29.3
10	Declining	Turnover time	No	4	0.24	166.98	6.40	16.5	0.24	166.99	6.41	16.5
12	Declining	Range size	No	6	0.28	176.93	16.35	8.50	0.63	198.55	37.97	0.00
9	Declining	None	No	4	0.19	177.08	16.50	1.10	0.18	177.12	16.54	1.00
14	Declining	Breeding system	No	5	0.20	179.54	18.96	2.00	0.27	179.73	19.15	2.00
11	Declining	Dispersal mode	No	7	0.39	181.82	21.24	1.60	0.65	193.61	33.03	0.00
13	Declining	Max ht	No	4	0.53	187.16	26.58	4.80	0.48	186.98	26.40	4.80
2	Constant	Turnover time	No	3	0.73	196.36	35.78	0.70	0.73	196.36	35.78	0.70
1	Constant	None	No	3	0.73	198.83	38.25	0.70	0.74	198.83	38.25	0.70
5	Constant	Max ht	No	3	0.78	199.59	39.01	0.01	0.78	199.59	39.01	0.01
3	Constant	Dispersal mode	No	6	0.74	200.05	39.47	0.00	0.74	200.09	39.51	0.00
4	Constant	Range size	No	5	0.78	201.79	41.21	0.00	0.77	200.17	39.59	0.00
6	Constant	Breeding system	No	4	0.75	202.02	41.44	0.01	0.75	202.02	41.44	0.01
8	Constant	Max ht	Yes	6	0.78	205.90	45.32	0.00	0.78	205.06	44.48	0.00
7	Constant	Turnover time	Yes	6	0.71	206.27	45.69	0.01	0.71	206.27	45.69	0.01
16	Declining	Max ht	Yes	7	0.62	212.55	51.97	22.5	0.88	258.10	97.52	14.3

Models are based on either a constant rate or exponentially declining rate of diversification which either include or exclude a range of ecological factors. Models ordered by AICc values; ΔAICc values calculated in relation to the best model. Model numbers refer to R code in Supporting Information. Pagel's λ varies from close to 1 (strong phylogenetic dependence of residuals) to small, negative values (negative correlation of residuals with phylogeny).

Overall, models that incorporate a decline in diversification rates over time provided a superior fit to the data compared to models with a constant rate of diversification (Table 1). This pattern emerges because there is no correlation between clade age and species richness in these data; an exponentially declining model of diversification provides a better fit in this case (Fig. 2). The best predictions of current species richness were achieved by a model that incorporated an exponential decline in diversification rates over time and a family-specific relationship between turnover rate and the initial rate of diversification (Fig. 3a). Allowing the effect of turnover rate on the diversification rate to vary among lineages improved predictions of the relative species richness of different families (e.g. low in the Moraceae and high in the Fabaceae; Fig. 3a). Nevertheless, the trend for higher species richness in clades with fast turnover times is found in several families (e.g. *Inga*, *Tachigali*, Fabaceae; *Guatteria,* Annonaceae; *Cecropia*, Urticaceae; Fig. 3).

We tested whether our final model was sensitive to uncertainty in the estimates of intrinsic mortality rates by running an additional model where the predictions were weighted by the uncertainty in these values (1/log(variance), Table S1). The relationship between observed and predicted values was very similar with this model suggesting that our results are not sensitive to uncertainty in this parameter (Fig. S2).

The value of Pagel's λ, which measures the degree of phylogenetic correlation among the residuals of these relationships, varied widely depending on the traits that were included in the diversification model (Table 1). Perhaps unsurprisingly, the smallest values of Pagel's λ were obtained with the best-fitting model where the parameters were allowed to vary among different branches of the phylogeny (Model 15, Table 1). Fitting the model separately for different lineages is likely to have reduced the phylogenetic signal in the residuals.

## Discussion

Our results demonstrate that short turnover times are linked to higher diversification rates and higher levels of species richness among multiple clades of tropical rain forest canopy trees. This result is found in several lineages and is robust to different underlying models of the diversification process, variation in relative extinction rates and uncertainty in estimates of turnover time, and remains significant after accounting for the phylogenetic dependence of different clades.

Fast demographic rates within tropical trees are related to a range of traits, such as rapid resource acquisition, high dispersal ability, fast growth in size and short lifespans (Turner 2001) and several of these attributes may drive the observed patterns. However, a link between fast turnover and short generation times may be a particularly important mechanism that drives this relationship. Mean generation time is the sum of the number of pre-reproductive years and the turnover time of reproductive individuals (Dillingham 2010). Inferring patterns of generation times from our measure of turnover times of trees ≥ 10 cm dbh requires two sets of assumptions related to patterns of reproduction and the time between seed dispersal and reproductive maturity. In terms of reproduction, the key assumptions are constant rates of tree survival and fecundity after the age of first reproduction (Dillingham 2010). For tropical forest canopy trees, these assumptions appear to be reasonable: the few data available on reproductive output are consistent with a minimum reproductive size of 10 cm diameter and constant reproductive output above this threshold. For example, for 12 species of trees with maximum height ≥ 15 m in Panama, the average minimum diameter for reproduction was 14.8 cm and reproduction did not decline at large sizes (Wright *et al.* 2005), and more generally mortality rates remain relatively constant with increasing size above 10 cm dbh (Rüger *et al.* 2011).

The second suite of assumptions requires considering the age of trees when they reach 10 cm diameter. We therefore estimated the passage time of different life-history stages from the literature, and compared estimates of total generation time with the intrinsic turnover times of trees ≥ 10 cm diameter for these clades (Appendix S3, Fig. S3). Although data are sparse, we found that the large and variable contribution of the lifespan of trees ≥ 10 cm diameter to estimates of total generation time suggests that this quantity is correlated with variation in generation time among these clades (Appendix S3).

Fast demographic traits could promote both the mechanisms – high speciation and low extinction rates – which lead to rapid diversification (Marzluff & Dial 1991). The capacity of populations to increase rapidly allows clades to undergo more rapid selection as new habitats and different resources become available, and to have faster rates of molecular evolution (Smith & Donoghue 2008). Both these processes may promote more rapid speciation, regardless of whether this is driven ultimately by vicariance, isolation due to long-distance dispersal or habitat specialization. Furthermore, shorter turnover times may also provide greater resilience to disturbances that cause extinction, such as climatic variation over interglacial cycles, by allowing successful migration to habitats with suitable environmental conditions and a greater ability to recolonize areas following such events.

The results of this study are broadly consistent with the few previous studies that have examined the relationship between diversification and demographic traits among woody plants. For example, Marzluff & Dial (1991) found negative, but non-significant, correlations between the age of first reproduction and total species richness for 10 gymnosperm and 19 angiosperm groups of North American trees, and Verdu (2002) found a significant negative correlation between genus species richness and age at maturity across 174 genera of mostly North American trees and shrubs. However, this is the first study of the correlates of diversification to focus on species-rich tropical forest trees and the first to develop comparative tests of different diversification models using a range of traits. This study is also the first to use directly measured, demographic data from permanent plots to estimate demographic traits: previous studies have used published data from the forestry literature, which are generally limited to species of commercial interest (Marzluff & Dial 1991; Verdu 2002). In contrast, the recent expansion of permanent plot networks in the tropics (e.g. Lopez-Gonzalez *et al.* 2009) now provides an opportunity to explore the role of life-history traits in determining evolutionary patterns across a wide range of clades in this biome.

Although specific trajectories of diversification will likely vary among clades, our study provides support for a general model of diversification where rates decline, rather than remain constant, over time (Table 1). This kind of model, and the limits to diversity that it implies within specific clades and regions (Fig. 2, Rabosky 2009a), has been proposed to explain the lack of correlation between clade age and species richness observed at some scales in some taxonomic groups (Rabosky *et al.* 2012) as well as the similar levels of diversity in different families of tropical plants on different continents (Ricklefs & Renner 2012). The precise mechanisms that determine this kind of pattern remain uncertain and debated (Wiens 2011; Rabosky *et al.* 2012), but processes that might contribute within individual clades include explosive radiations resulting from the emergence of novel ecological opportunities or morphological innovation (Rabosky *et al.* 2012), and/or ‘carrying capacities’ in the number of species that different regions can support (Rabosky 2009a).

Where diversification rates vary over time, interpreting how ecological covariates might influence the diversification process is more challenging than in constant rate models. In the broadest sense and regardless of the underlying model of diversification, significant relationships between ecological factors and the total species richness of different clades suggest that, integrated over the age of the clade, those factors must have promoted speciation and/or reduced extinction rates (Rabosky 2009a). However, our model formulation suggests more specifically that intrinsic factors affecting the *initial* rate of diversification of a clade is one way ecological covariates might influence the total levels of diversification that clades achieve. Similarly, Rabosky (2009b) found that relating range size to the initial rate of diversification improved predictions of species richness across 88 tribes of birds compared to a model without ecological covariates. In the context of Amazonia, this framework suggests that clades with fast demographic traits may be able to exploit specific opportunities for diversification more rapidly following geological events that create novel habitats, such as the deposition or exposure of particular edaphic conditions (Hoorn *et al.* 2010). Overall, this interpretation emphasises the close links between historical processes and the intrinsic traits of different lineages in generating observed patterns of diversity.

Some ecological factors that are often associated with patterns of diversification in plants, such as range size and maximum height (Givnish 2010), were not significant in this study. However, the focus of this study on Neotropical canopy trees of at least 10 cm dbh meant that many of the clades had similar values for these traits, and this study therefore excluded woody understory plants that contain some species-rich genera (e.g. *Psychotria*, Rubiaceae). Range size marginally improved predictions of diversification under an exponential model, with clades with large range sizes containing more species (Table 2). This factor may be a more important factor explaining diversification in larger scale, pantropical analyses.

**Figure 4:**
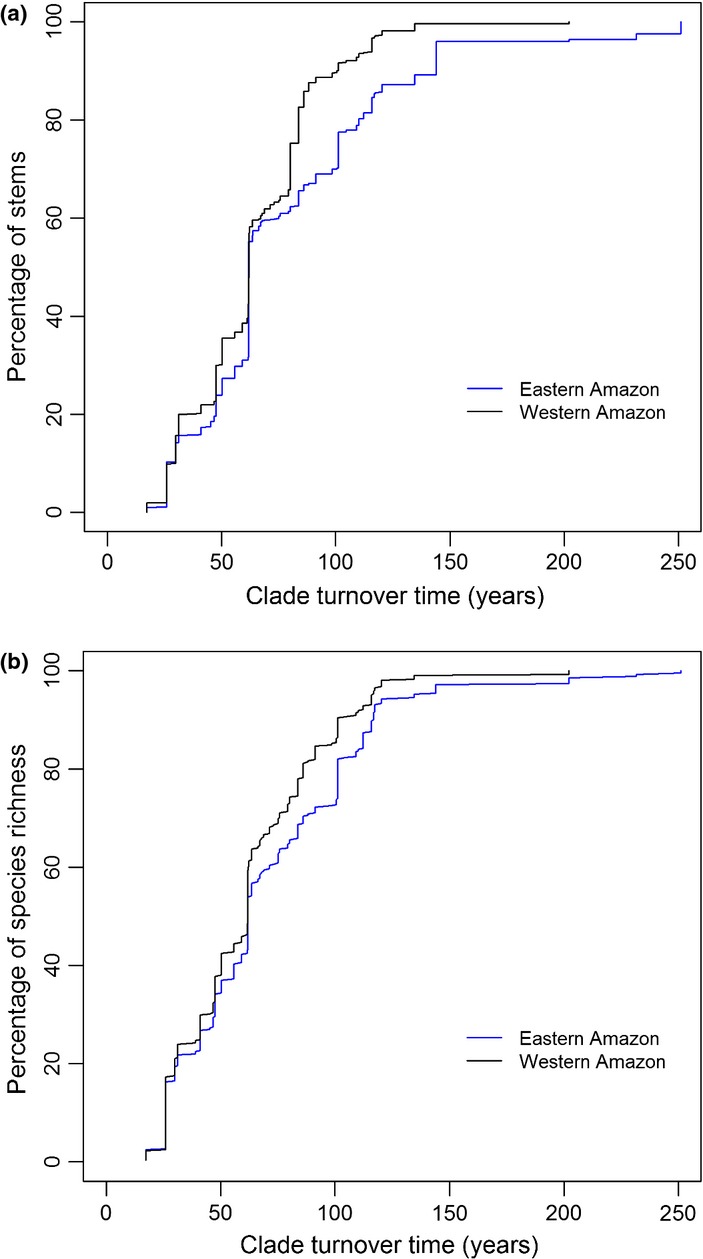
(a) The cumulative abundance of 51 clades of tropical trees with different intrinsic turnover times, in western (black) and eastern (blue) Amazon forests. (b) The contribution of clades with different intrinsic turnover times to the species richness of forests in western and eastern Amazon forests.

Although our focus here is on understanding variation in species richness among clades of tropical trees, our results also have implications for understanding community-level patterns of diversity within Amazonia. In particular, high rates of diversification of fast turnover clades may have contributed to generating the particularly high diversity of western Amazon forests (Gentry 1988). Among the 51 clades in this study, those with intrinsically fast turnover times make a greater contribution to the stems and species richness of western Amazon forests compared to forests in eastern Amazonia and the Guiana Shield (Fig. 4). Genera with fast turnover times also contribute more stems and species to western Amazon forests across all 150 genera with > 100 stems in the RAINFOR plot network (Table S3, Fig. S4) and the turnover times of this larger group of genera are also correlated with their species richness (Fig. S5). In addition, species-rich clades with intrinsically short turnover times are more diverse in western Amazonia: the diversity of both *Inga* and *Guatteria* is approximately a third higher in the plots in these forests (per 100 stems: *Inga*, western Amazonia, 37 species; eastern Amazonia, 28 species; *Guatteria*, western Amazonia, 19 species; eastern Amazonia, 15 species). These patterns suggest that the environmental conditions associated with high rates of tree mortality in western Amazonia (Quesada *et al.* 2012) have favoured lineages with intrinsically shorter turnover times. In turn, these lineages may have shown high *in situ* diversification rates in response to historical events such as climatic shifts and deposition of novel edaphic conditions (Hoorn *et al.* 2010). A complex interaction of both intrinsic and extrinsic factors may have therefore generated the high diversity of this region.

The ecological trait of short turnover times is shared by some of the most species-rich groups of Amazonian trees, such as *Inga* and *Guatteria,* with ≈ 300 and ≈ 265 species respectively. Overall, our results indicate that ecological differences among clades of tropical trees have strongly influenced their diversification, and the high level of diversification in lineages with short turnover times has played a key role in generating the spectacular diversity of Amazonian forests.
