# Supplementary material for: Fast demographic traits promote high diversification rates of Amazonian trees
Source: Ecol Lett. 2014 Mar 3;17(5):527–36. doi: 10.1111/ele.12252 (PMC4285998; doi:10.1111/ele.12252)
Supplement: Supplementary file 1 — supplementary [file ele0017-0527-SD1.docx]

**Appendix S1: determinants of tree mortality rates**

Understanding the importance of environmental factors and intrinsic variation among clades for determining variation in plant traits, such as mortality rates, can be achieved by using mixed models (e.g. Fyllas *et al.* 2009). We initially explored the relative importance of the identity and location of trees on the probability of mortality using a mixed model with binomial errors using the *lmer* package (Bates *et al.* 2011) in R (R Development Core Team 2012). We used the state of each individual tree (alive/dead) at the final census as the response variable, and tested the effect of identity using clade and species as random factors, with species nested within clade. We simultaneously tested the effect of location using plot, and ‘plot cluster’ as additional random factors, with plot nested within ‘plot cluster’. ‘Plot clusters’ share the same three letter code in Table S2 and capture the importance of the broad-scale environmental gradients across Amazonia on stand-level mortality rates (Quesada *et al.* 2012). In contrast, the ‘plot’ level effect captures the variation in mortality rates among plots within sites, due to differences in topography or soil type. We analysed data from all 51 clades including identified species represented by at least 10 individuals to avoid overfitting; overall, this dataset included 30,440 trees in 347 identified species, spread amongst 57 plot clusters in 187 individual plots.

Differences among clades accounted for more than double the variation among species (variance components: clade, 0.213 (SD 0.461); species nested within clades, 0.101 (SD 0.318). Overall, species within the same clade therefore share similar mortality rates, whereas clades vary more widely. This finding supports the idea that there are significant differences in the intrinsic mortality rates of different clades. In terms of the effect of location, ‘plot cluster’ (variance component 0.543, SD 0.737), strongly outweighed the influence of individual plots (variance component 0.161, SD 0.402). Broad differences in climate and soils across Amazonia therefore have a larger effect on mortality rates than small-scale variation within sites.

**References**

Bates, D., Maechler, M. & Bolker, B. (2011) lme4: Linear mixed-effects models using S4 classes R package version 0.999375-42.

Fyllas, N.M., Patino, S., Baker T.R., Bielefeld Nardoto, G., Martinelli, L. A., Quesada, C.A., Paiva, R., Schwarz, M., Horna, V., Mercado, L.M., Santos, A., Arroyo, L., Jímenez, E.M., Luizao, F., Neill, D.A., Silva, N., Prieto, A., Rudas, A., Silveira, M., Vieira, I.C.G., Lopez Gonzalez, G., Malhi, Y., Phillips, O.L. & Lloyd, J. (2009) Basin-wide variations in foliar properties of Amazonian forest: phylogeny, soils and climate. *Biogeosciences,* 6**,** 2677-2708.

Quesada C.A., Phillips O.L., Schwarz M., Czimczik C.I., Baker T.R., Patiño S., Fyllas N.M., Hodnett M., Herrera R., Almeida S., Alvarez Dávila E., Arneth A., Arroyo L., Chao K.-J., Dezzeo N., Erwin T., di Fiore A., Higuchi N., Honorio Coronado E., Jiménez E.M., Killeen T., Lezama A.T., Lloyd G., López-González G., Luizão F.J., Malhi Y., Monteagudo A., Neill D.A., Núñez Vargas P., Paiva R., Peacock J., Peñuela M.C., Peña Cruz A., Pitman N.C.A., Priante Filho N., Prieto A., Ramírez H., Rudas A., Salomão R., Santos A.J.B., Schmerler J., Silva N., Silveira M., Vásquez R., Vieira I., Terborgh J. & Lloyd J. (2012). Basin-wide variations in Amazon forest structure and function are mediated by both soils and climate *Biogeosciences*, 9, 2203-2246.

R Development Core Team (2012). *R: A Language and Environment for Statistical Computing*. R Foundation for Statistical Computing, Vienna.
