# Supplementary material for: Fast demographic traits promote high diversification rates of Amazonian trees
Source: Ecol Lett. 2014 Mar 3;17(5):527–36. doi: 10.1111/ele.12252 (PMC4285998; doi:10.1111/ele.12252)
Supplement: Supplementary file 2 — supplementary [file ele0017-0527-SD2.docx]

**Appendix S2: diversification analysis**

We fit our models of diversification using maximum likelihood assuming a multivariate normal distribution of errors with a correlation structure chosen to reflect the underlying phylogenetic relationships among taxa (Bolker 2008; Revell 2010). The expected correlation matrix was based on the shared branch lengths of the phylogenetic relationships among taxa under a Brownian motion model of trait evolution (Paradis 2012). We modelled the error structure using a transformed version of this correlation matrix that incorporates Pagel’s *λ* (Pagel 1999) as a measure of the strength of the phylogenetic signal in the residuals. For the key trait in this study - turnover time - this model of evolution (AICc 538.6) out-performed a pure Brownian motion model (AICc = 562.83) and the Ornstein Uhlbeck model (AICc = 541.56; models fit using the *fitContinuous* function in *geiger* (Harmon *et al.* 2008) using R (R Development Core Team 2012).

We developed predictions of species richness across clades based on solving equation (1) for different formulations of the diversification rate, *r*, and compared these with the observed values. We simultaneously estimated the parameters of the diversification models and Pagel’s *λ* as recommended by Revell (2010) using the *optim* function in R. For all models using individual ecological variables, we used the Nelder-Mead function for optimisation (Nelder and Mead 1965); for the model incorporating variation in *r* among families, we obtained improved results by first estimating the parameters of the diversification model using Nelder Mead with Pagel’s *λ =* 0, and then refining these parameters and simultaneously estimated Pagel’s *λ*, using simulated annealing using the ‘SANN’ method (Belisle 1992) with 5 x 10^5^ iterations.

#R code to perform diversification analysis based on an exponential model of diversification where the effect of turnover times (TT) varies among families (model 15)

#estimate underlying error structure, where ‘*phylogeny*’ is the phylogenetic relationships of the study taxa, of class *phylo*.

#uses *ape* package (Paradis *et al.* 2004)

errors<-vcv(phy=phylogeny, model="Brownian", corr=T)

#set extinction rate

ext=0

#fit model where *p* is a vector of model parameters and *p[7]* estimates Pagel’s *λ.*; *t,* is clade age in Ma; *a***,** whether the age relates to a stem (*a*=1) or crown (*a*=2) age; *ext* is the relative extinction rate (0 or 0.9), and *b(n)* is a suite of dummy variables indicating membership of *n* families by different clades.

#initial parameter values estimated using model excluding correlated error structure

normNLL = function(p) {

y.species = ((1-ext)*((p[1]*(1/log(TT))*b1)+

(p[2]*(1/log(TT))*b2)+(p[3]*(1/log(TT))*b3)

+(p[4]*(1/log(TT))*b4))

*(1/p[5])*(1-exp(-p[5]*t)))+log(a)

-sum(dmvnorm(log(species),mean=y.species,

sigma=p[6]*(p[7]*(errors-diag(diag(errors)))+diag(diag(errors))),

log=T))}

out<-optim(p=c(4.9,7.7,9.8,5.9,0.04,2.04,0.5),normNLL,

control=list(maxit=500000),method="SANN")

Other models used to estimate species richness were formulated as:

| Model | Type | Description | Family-specific? | Form |
| --- | --- | --- | --- | --- |
| 1 | *Constant* | *No traits* | No | (p[1]*(1-ext)*t)+log(a) |
| 2 | *Constant* | *Turnover* | No | (p[1]*(1-ext)*(1/log(TT))*(t))+log(a) |
| 3 | *Constant* | *Dispersal* | No | (1-ext)*((p[1]*Dispersal1)+(p[2]*Dispersal2)+…))*(t))+log(a) |
| 4 | *Constant* | *Range size* | No | (1-ext)*((p[1]*Range1)+(p[2]*Range2)+…))*(t))+log(a) |
| 5 | *Constant* | *Max height* | No | (p[1]*(1-ext)*(1/Ht)*(t))+log(a) |
| 6 | *Constant* | *Breeding system* | No | (1-ext)*((p[1]*Breeding1)+(p[2]*Breeding2))*(t))+log(a) |
| 7 | *Constant* | *Turnover* | Yes | (1-ext)*((p[1]*(1/log(TT))*b1)+(p[2]*(1/log(TT))*b2)…)*t)+log(a) |
| 8 | *Constant* | *Max height* | Yes | (1-ext)*((p[1]*(1/(Ht))*b1)+(p[2]*(1/(Ht))*b2)+…*t)+log(a) |
| 9 | *Exponential* | *No traits* | No | (p[1]*(1-ext)*(1/p[2])*(1-exp(-p[2]*t)))+log(a) |
| 10 | *Exponential* | *Turnover* | No | (p[1]*(1-ext)*(1/log(TT))*(1/p[2])*(1-exp(-p[2]*t)))+log(a) |
| 11 | *Exponential* | *Dispersal* | No | ((1-ext)*((p[1]*Dispersal1)+(p[2]*Dispersal2)+…))*(1/p[5])*(1-exp(-p[5]*t)))+log(a) |
| 12 | *Exponential* | *Range size* | No | ((1-ext)*((p[1]*Range1)+(p[2]*Range2)+…)*(1/p[5])*(1-exp(-p[5]*t)))+log(a) |
| 13 | *Exponential* | *Max height* | No | (p[1]*(1-ext)*(1/(Ht))*(1/p[2])*(1-exp(-p[2]*t)))+log(a) |
| 14 | *Exponential* | *Breeding system* | No | ((1-ext)*((p[1]*Breeding2)+(p[2]*Breeding2))*(1/p[3])*(1-exp(-p[3]*t)))+log(a) |
| 15 | *Exponential* | *Turnover* | Yes | ((1-ext)*((p[1]*(1/log(TT))*b1)+(p[2]*(1/log(TT))*b2))+…)  *(1/p[5])*(1-exp(-p[5]*t)))+log(a) |
| 16 | *Exponential* | *Max height* | Yes | ((1-ext)*((p[1]*(1/Ht)*b1)+(p[2]*(1/Ht)*b2)+…)*(1/p[5])*(1-exp(-p[5]*t)))+log(a) |

In these models, *Dispersal*, *Range* and *Breeding* are suites of dummy variables indicating membership of different categories of these traits for different clades (Table 1).

**References**

Belisle, C. J. P. (1992) Convergence theorems for a class of simulated annealing algorithms on *Rd*. J. Applied Probability, **29**, 885–895.

Bolker B.M. (2008). *Ecological models and data in R*. Princeton University Press.

Harmon L.J., Weir J.T., Brock C.D., Glor R.E. & Challenger W. (2008). GEIGER: investigating evolutionary radiations. *Bioinformatics*, 24, 129-131.

Nelder, J. A. and Mead, R. (1965) A simplex algorithm for function minimization. Computer Journal **7**, 308–313.

Pagel M. (1999). Inferring the historical patterns of biological evolution. *Nature*, 401, 877-884.

Paradis E. (2012). *Analysis of Phylogenetics and Evolution with R*. Springer.

Paradis E., Claude J. & Strimmer K. (2004). APE: analyses of phylogenetics and evolution in R language. *Bioinformatics*, 20, 289-290.

R Development Core Team (2012). *R: A Language and Environment for Statistical Computing*. R Foundation for Statistical Computing, Vienna.

Revell L.J. (2010). Phylogenetic signal and linear regression on species data. *Methods in Ecology and Evolution*, 1, 319-329.
