# Supplementary material for: Fast demographic traits promote high diversification rates of Amazonian trees
Source: Ecol Lett. 2014 Mar 3;17(5):527–36. doi: 10.1111/ele.12252 (PMC4285998; doi:10.1111/ele.12252)
Supplement: Supplementary file 3 — supplementary [file ele0017-0527-SD3.docx]

**Appendix S3: turnover and generation times**

An important uncertainty in the relationship between total generation time and the turnover times of trees ≥10 cm dbh is the time taken for individuals to reach this minimum size threshold. We examined whether the magnitude and variation in passage times of trees in smaller size classes might remove any relationship between generation time and the turnover times of trees ≥10 cm dbh.

We estimated the mean and variability of passage times for sapling to 10 cm diameter tree, seedling to sapling and seed to seedling transitions based on published data for neotropical trees. For the passage time of tree 0-10 cm dbh, we used data from tree ring analysis of six species that encompass fast-growing (e.g. *Tachigali vasquezii*) to slow-growing, shade tolerant species (e.g. *Peltogyne* cf. *heterophylla*) from rain forest in Bolivia (Brienen & Zuidema 2006). Ages from tree ring analysis of large trees are particularly useful for this calculation as they record the growth of trees that were ultimately successful in reaching the canopy; calculations based on average growth rates from permanent plots would be biased downwards by slow-growing individuals that died before reproducing. Average age of these species at 10 cm dbh was 30.5 ± 10.8 (SD) years (Brienen & Zuidema 2006). Passage times to reach 1.3 m height (dbh) were estimated from data for six non-pioneer species from Costa Rica (Clark & Clark 2001) as 16.63 ± 10.8 years (0-50 cm height) and 9.26 ± 10.8 years (50-130 cm height). We assumed that the time for seedling emergence was one year across all non-pioneer species. The only clade we treated differently was *Cecropia*. Seeds for pioneer species, such as *Cecropia*, may remain dormant for extended periods of time (Dalling & Brown 2009); we therefore modelled the time for seedling emergence as an exponential distribution with a rate parameter of 0.25. This distribution occasionally generates seed emergence times of around 30 years, consistent with carbon dated ages of some viable seeds of pioneer species extracted from the soil seed bank in Panama (Dalling & Brown 2009). After emergence, *Cecropia* grows fast; we estimated passage times as 3.65 ± 0.45 years (0-50 cm height) and 5.84 ± 0.72 years (50-130 cm height) based on Clark and Clark (2001).

To estimate the total generation times of the 51 clades in this study, we sampled from the distribution of passage times for each life history stage, including our own data on the mean and variability in the intrinsic turnover times of trees ≥10 cm dbh, and summed the results. We compared these estimates with the turnover times of trees ≥10 cm dbh and calculated the mean and 95% confidence limits of the slope of this relationship based on resampling.

Overall, average estimates of total generation time are correlated with the turnover time of trees ≥10 cm dbh, and the slope of this relationship is significantly greater than zero (Fig. S3; mean slope 1.71; upper 95 % confidence limit 5.56; lower 95 % confidence limit 0.39). After accounting for the uncertainty in passage times of other life history stages, the relationship between the turnover time of trees ≥10 cm dbh and overall generation time remains because the turnover time of trees ≥10 cm dbh is a large and variable component of the calculation. We acknowledge that the lack of data means that many uncertainties remain (although these uncertainties include possible correlations between passage times across different life history stages that might strengthen the relationship). However, this consideration of the available data suggest that a correlation between total generation times and the turnover times of trees ≥10 cm dbh is not unreasonable, as the passage times for this life history stage are an important contributor to variation in estimates of generation time across multiple clades.

**References**

Brienen R.J. & Zuidema P.A. (2006). Lifetime growth patterns and ages of Bolivian rain forest trees obtained by tree ring analysis. *Journal of Ecology*, 94, 481-493.

Clark D.A. & Clark D.B. (2001). Getting to the canopy: tree height growth in a neotropical rain forest. *Ecology*, 82, 1460-1472.

Dalling J.W. & Brown T.A. (2009). Long‐term persistence of pioneer species in tropical rain forest soil seed banks. *The American Naturalist*, 173, 531-535.
