# Supplementary material for: Fast demographic traits promote high diversification rates of Amazonian trees
Source: Ecol Lett. 2014 Mar 3;17(5):527–36. doi: 10.1111/ele.12252 (PMC4285998; doi:10.1111/ele.12252)

**Fig. S1.** Phylogenetic relationships amongst 51 clades of neotropical tree used to account for the phylogenetic non-independence of data in the analysis of diversification.


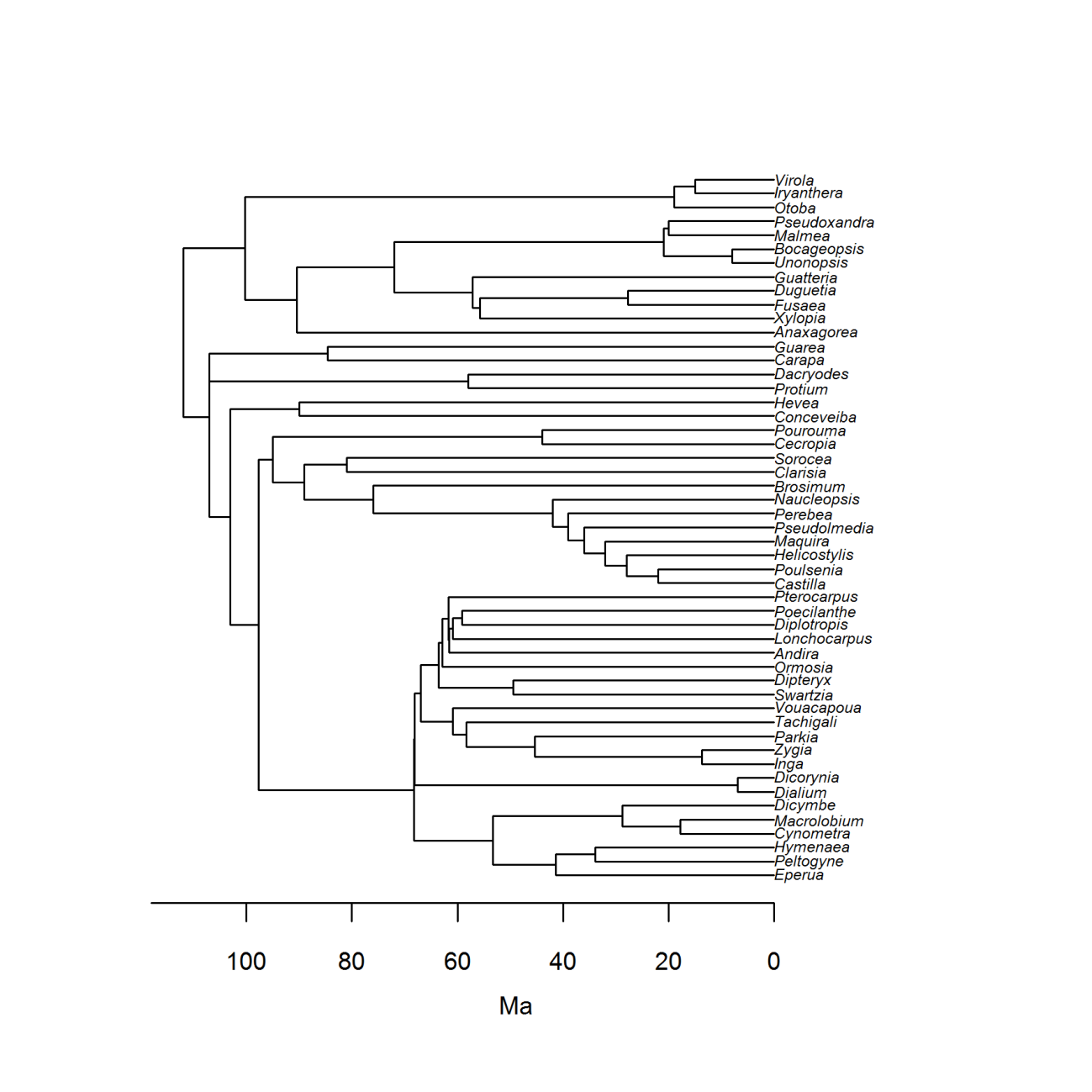

Supplement: Supplementary file 4 — supplementary [file ele0017-0527-SD4.docx]
