# Supplementary material for: Fast demographic traits promote high diversification rates of Amazonian trees
Source: Ecol Lett. 2014 Mar 3;17(5):527–36. doi: 10.1111/ele.12252 (PMC4285998; doi:10.1111/ele.12252)

**Fig. S2**. Relationships between observed species richness and predictions based on an exponentially declining model of diversification where the initial rate of diversification is proportional to the intrinsic turnover time of the clade. (a) predictions from final model of main manuscript (Table 1), (b) predictions with the same model, but observations weighted by the variance in intrinsic turnover time for each clade.


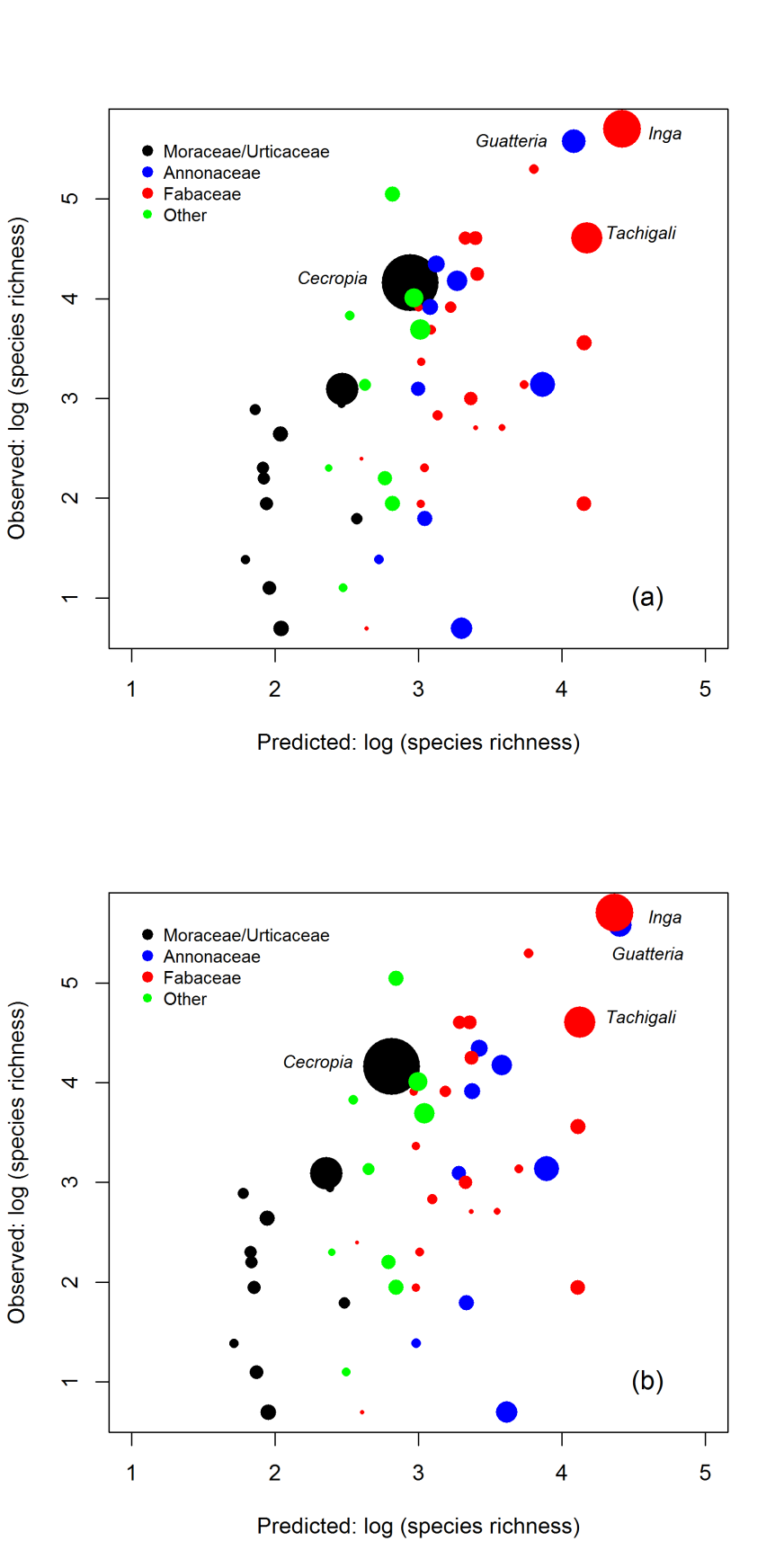

Supplement: Supplementary file 5 — supplementary [file ele0017-0527-SD5.docx]
