# Supplementary material for: Fast demographic traits promote high diversification rates of Amazonian trees
Source: Ecol Lett. 2014 Mar 3;17(5):527–36. doi: 10.1111/ele.12252 (PMC4285998; doi:10.1111/ele.12252)

**Fig S3**. Relationship between the intrinsic turnover time of trees ≥10 cm dbh for 51 clades of tree and estimates of total generation times, including estimates of the passage times for the transitions through the seed, sapling and small-tree life history stages.


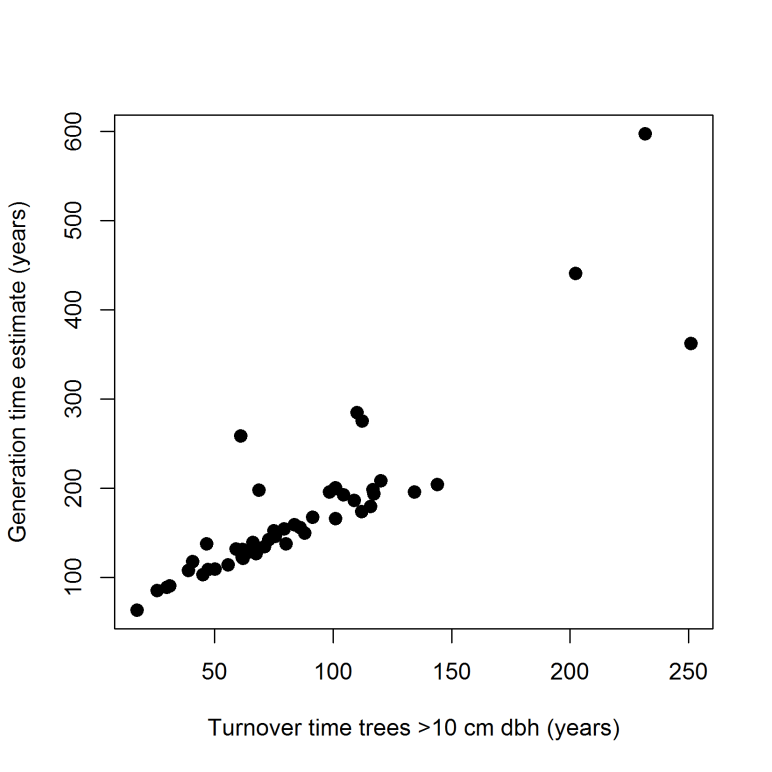

Supplement: Supplementary file 6 — supplementary [file ele0017-0527-SD6.docx]
