# Supplementary material for: Fast demographic traits promote high diversification rates of Amazonian trees
Source: Ecol Lett. 2014 Mar 3;17(5):527–36. doi: 10.1111/ele.12252 (PMC4285998; doi:10.1111/ele.12252)

**Fig. S4.** Contribution of 150 genera with different average turnover times to the overall species richness of western and eastern Amazonian forests and forests on the Guiana Shield.


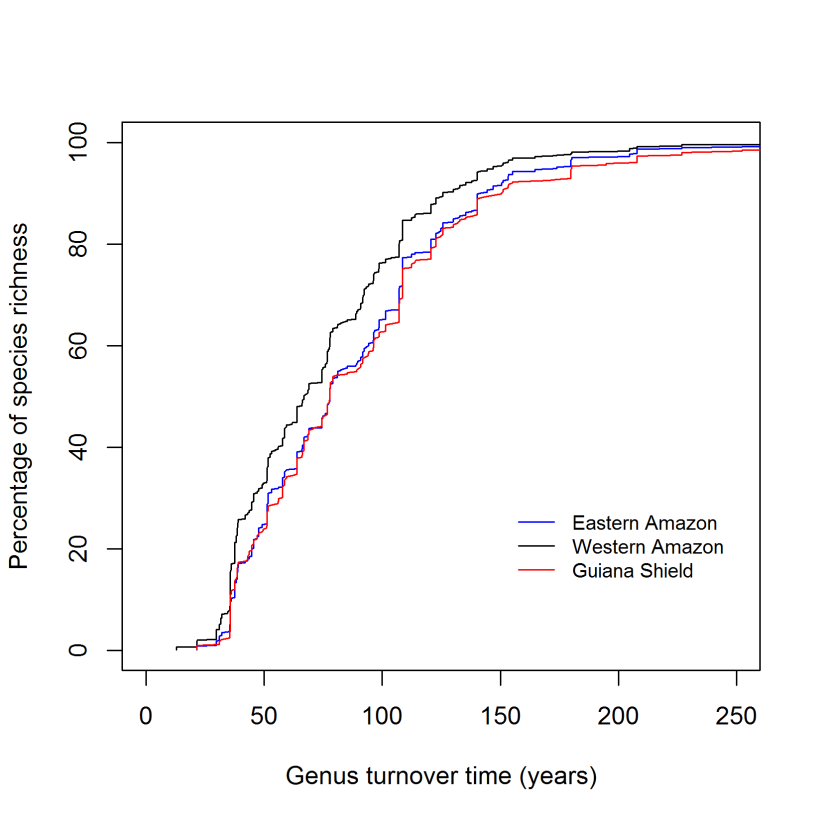

Supplement: Supplementary file 7 — supplementary [file ele0017-0527-SD7.docx]
