# Supplementary material for: Fast demographic traits promote high diversification rates of Amazonian trees
Source: Ecol Lett. 2014 Mar 3;17(5):527–36. doi: 10.1111/ele.12252 (PMC4285998; doi:10.1111/ele.12252)

**Fig. S5.** Relationship between species richness and average turnover time for clades included in diversification analyses (solid circles), and all additional genera with >100 stems where average turnover time can be estimated using the forest plot data (open circles). Regression line shows relationship for all groups.


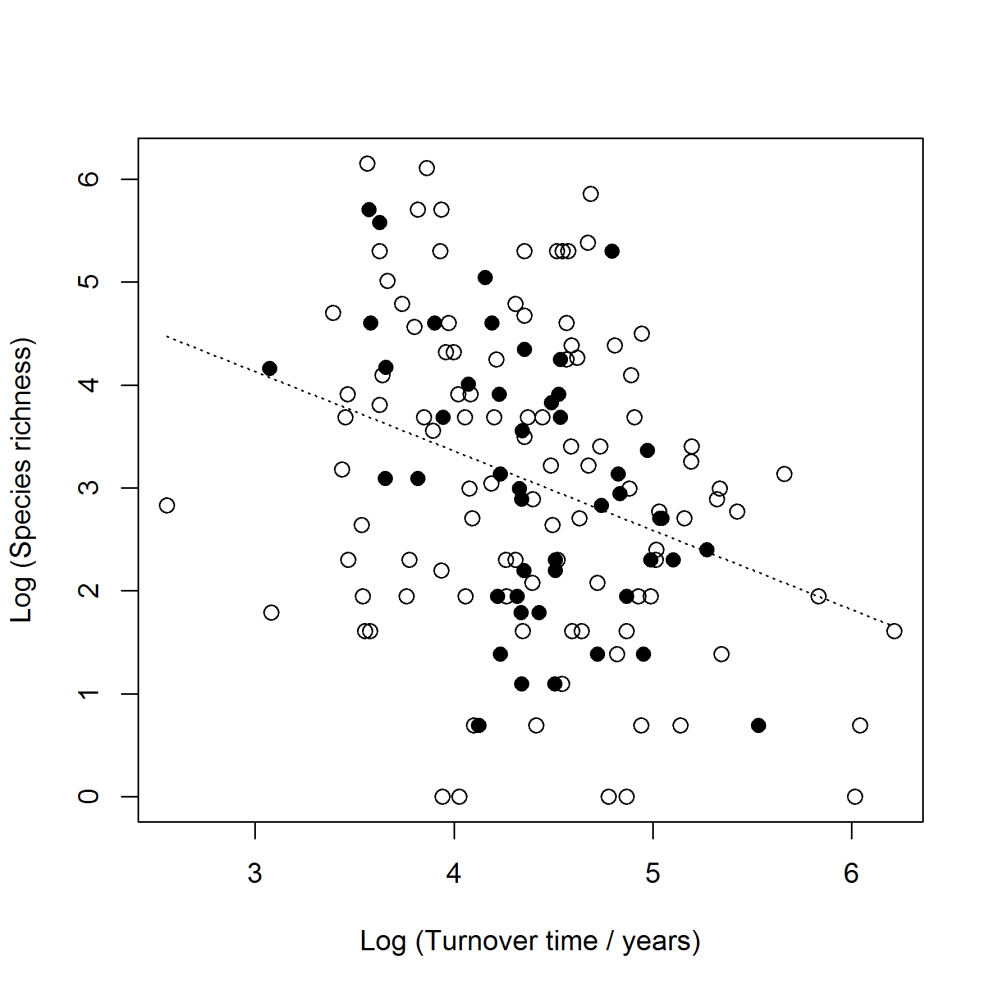

Supplement: Supplementary file 8 — supplementary [file ele0017-0527-SD8.docx]
