# Supplementary material for: Fast demographic traits promote high diversification rates of Amazonian trees
Source: Ecol Lett. 2014 Mar 3;17(5):527–36. doi: 10.1111/ele.12252 (PMC4285998; doi:10.1111/ele.12252)
Supplement: Supplementary file 9 — supplementary [file ele0017-0527-SD9.docx]

**Table S1.** Ages and ecological properties of 51 clades used for calculation of diversification rates: crown/stem ages (Ma), species richness (from Pennington *et al.* 2004; The Plant List 2010), intrinsic turnover time and uncertainty (weight: 1/log(variance) of estimate of intrinsic turnover time), maximum height, range size, predominant dispersal mode and breeding system. Dispersal mode: 1, explosive/unassisted; 2, arboreal or ground dwelling mammal; 3, bat or bird; 4, water; 5, wind. Range: P, pantropical; N, Neotropical S, South American; G, Guiana Shield. Breeding system: M, monoecious; D, dioecious. References: (1) Couvreur *et al.* (2010), (2) Weeks *et al.* (2005), (3) Davis *et al.* (2005), (4) Simon *et al.* (2009), (5) Muellner *et al.* (2006), (6) Zerega *et al.* (2005), (7) Erkens *et al.* (2007).

|  |  |  |  |  |  |  |  |  |  |  |  |
| --- | --- | --- | --- | --- | --- | --- | --- | --- | --- | --- | --- |
| Family | Genus | Stem age | Crown age | Ref | Species | Intrinsic  turnover time | Weight | Max ht | Dispersal | Range | Breeding system |
|  |  | Ma | Ma |  | no. | years |  | m |  |  |  |
| Annonaceae | *Anaxagorea* | 90.44 | 63 | 1 | 23 | 39.02 | 1.56 | 14 | 1 | P | M |
| Annonaceae | *Bocageopsis* | 5.98 |  | 1 | 4 | 101.12 | 1.37 | 20 | 3 | S | M |
| Annonaceae | *Duguetia* | 30.64 |  | 1 | 77 | 55.76 | 4.55 | 17 | 3 | N | M |
| Annonaceae | *Fusaea* | 30.64 |  | 1 | 2 | 45.07 | 4.43 | 23 | 3 | S | M |
| Annonaceae | *Guatteria* | 55.83 | 11.4 | 1,7 | 265 | 40.82 | 1.27 | 25 | 3 | N | M |
| Annonaceae | *Malmea* | 19.99 |  | 1 | 6 | 62.09 | 3.84 | 31 | 3 | S | M |
| Annonaceae | *Pseudoxandra* | 15.09 |  | 1 | 22 | 66.20 | 1.72 | 21 | 3 | S | M |
| Annonaceae | *Unonopsis* | 7.94 |  | 1 | 50 | 59.09 | 1.66 | 16 | 3 | N | M |
| Annonaceae | *Xylopia* | 49.98 |  | 1 | 65 | 46.67 | 1.10 | 22 | 3 | P | M |
| Burseraceae | *Dacryodes* | 38 |  | 2 | 46 | 100.59 | 1.37 | 23 | 3 | N | D |
| Burseraceae | *Protium* | 52.5 |  | 2 | 155 | 61.86 | 2.47 | 22 | 3 | N | D |
| Euphorbiaceae | *Conceveiba* | 72 |  | 3 | 9 | 66.96 | 2.07 | 20 | 1 | P | D |
| Euphorbiaceae | *Hevea* | 85 |  | 3 | 10 | 134.42 | 4.19 | 30 | 1 | S | M |
| Fabaceae | *Andira* | 17.51 |  | 4 | 29 | 116.83 | 1.83 | 24 | 3 | P | M |
| Fabaceae | *Cynometra* | 12.93 |  | 4 | 70 | 67.62 | 4.15 | 40 | 3 | P | M |
| Fabaceae | *Dialium* | 10.9 |  | 4 | 40 | 104.49 | 1.58 | 33 | 3 | P | M |
|  |  |  |  |  |  |  |  |  |  |  |  |
| Family | Genus | Stem age | Crown age | Ref | Species | Intrinsic  turnover time | Weight | Max ht | Dispersal | Range | Breeding system |
|  |  | Ma | Ma |  | no. | years |  | m |  |  |  |
| Fabaceae | *Dicorynia* | 10.9 |  | 4 | 2 | 231.73 | 0.88 | 50 | 5 | G | M |
| Fabaceae | *Dicymbe* | 12 |  | 4 | 17 | 98.41 | 1.39 | 35 | 2 | G | M |
| Fabaceae | *Diplotropis* | 20.27 |  | 4 | 7 | 117.14 | 2.05 | 32 | 5 | S | M |
| Fabaceae | *Dipteryx* | 26.44 |  | 4 | 10 | 112.22 | 0.99 | 36 | 3 | N | M |
| Fabaceae | *Eperua* | 12.32 | 7.34 | 4 | 15 | 144.01 | 4.90 | 40 | 1 | G | M |
| Fabaceae | *Hymenaea* | 24.67 | 9.67 | 4 | 15 | 202.24 | 1.02 | 32 | 2 | P | M |
| Fabaceae | *Inga* | 10 |  | 4 | 300 | 25.84 | 2.47 | 22 | 3 | N | M |
| Fabaceae | *Lonchocarpus* | 15.07 |  | 4 | 100 | 68.65 | 0.96 | 40 | 5 | P | M |
| Fabaceae | *Macrolobium* | 32 |  | 4 | 50 | 86.02 | 2.13 | 29 | 1 | N | M |
| Fabaceae | *Ormosia* | 40.62 |  | 4 | 100 | 75.07 | 1.64 | 17 | 2 | P | M |
| Fabaceae | *Parkia* | 45.5 | 6.2 | 4 | 35 | 63.37 | 2.49 | 38 | 3 | P | M |
| Fabaceae | *Peltogyne* | 28.8 | 12.55 | 4 | 23 | 112.09 | 3.88 | 40 | 5 | N | M |
| Fabaceae | *Poecilanthe* | 40.99 | 20.52 | 4 | 7 | 63.60 | 2.26 | 12 | 1 | S | M |
| Fabaceae | *Pterocarpus* | 16.66 |  | 4 | 20 | 71.19 | 2.67 | 24 | 5 | P | M |
| Fabaceae | *Swartzia* | 45.96 | 13.62 | 4 | 200 | 101.11 | 2.85 | 24 | 3 | N | M |
| Fabaceae | *Tachigali* | 4.65 |  | 4 | 100 | 31.21 | 2.64 | 29 | 5 | N | M |
| Fabaceae | *Vouacapoua* | 48.69 |  | 4 | 11 | 250.95 | 1.84 | 35 | 2 | G | M |
| Fabaceae | *Zygia* | 17.82 |  | 4 | 50 | 120.14 | 1.67 | 14 | 1 | N | M |
| Meliaceae | *Carapa* | 29.5 |  | 5 | 3 | 110.12 | 0.95 | 35 | 2 | N | M |
| Meliaceae | *Guarea* | 14.8 |  | 5 | 55 | 50.21 | 3.79 | 24 | 3 | P | D |
| Moraceae | *Brosimum* | 48 | 28 | 6 | 19 | 115.81 | 3.30 | 40 | 3 | N | D |
| Moraceae | *Castilla* | 22 |  | 6 | 3 | 72.92 | 2.01 | 33 | 3 | N | D |
| Moraceae | *Clarisia* | 65 | 49 | 6 | 6 | 88.05 | 3.45 | 30 | 3 | N | D |
| Moraceae | *Helicostylis* | 28 |  | 6 | 7 | 75.65 | 1.98 | 26 | 3 | N | D |
|  |  |  |  |  |  |  |  |  |  |  |  |
| Family | Genus | Stem age | Crown age | Ref | Species | Intrinsic  turnover time | Weight | Max ht | Dispersal | Range | Breeding system |
|  |  | Ma | Ma |  | no. | years |  | m |  |  |  |
| Moraceae | *Poulsenia* | 22 |  | 6 | 2 | 61.05 | 0.74 | 35 | 3 | N | M |
| Moraceae | *Pseudolmedia* | 36 |  | 6 | 10 | 80.16 | 7.10 | 33 | 3 | N | D |
| Moraceae | *Sorocea* | 59 |  | 6 | 14 | 61.65 | 3.19 | 12 | 3 | N | D |
| Myristicaceae | *Iryanthera* | 19 |  | 3 | 23 | 83.69 | 1.82 | 23 | 3 | S | D |
| Myristicaceae | *Otoba* | 17 |  | 3 | 7 | 61.91 | 1.86 | 32 | 3 | N | D |
| Myristicaceae | *Virola* | 17 |  | 3 | 40 | 47.36 | 2.56 | 27 | 3 | N | D |
| Urticaceae | *Cecropia* | 44 |  | 6 | 64 | 17.33 | 2.48 | 21 | 3 | N | D |
| Urticaceae | *Pourouma* | 44 |  | 6 | 22 | 30.04 | 3.10 | 24 | 3 | N | D |
