# Supplementary material for: Fast demographic traits promote high diversification rates of Amazonian trees
Source: Ecol Lett. 2014 Mar 3;17(5):527–36. doi: 10.1111/ele.12252 (PMC4285998; doi:10.1111/ele.12252)
Supplement: Supplementary file 10 — supplementary [file ele0017-0527-SD10.docx]

**Table S2**. Coordinates, plot area and region (WA, western Amazonia; EA, eastern Amazonia and Guiana Shield (GS); SA, southern Amazonia; NSA, northern South America) for 207 multiple census forest inventory plots used for calculating turnover times and 20 single census plots included in the calculation of the proportion of total species richness in genera with different turnover times.

| Code | Name | Region | Area  ha | First census | Last census | Lat.  dec. | Long.  dec. |
| --- | --- | --- | --- | --- | --- | --- | --- |
| AGJ-01 | Aguajal | WA | 2.25 | 1993.759 | 2003.671 | -11.886 | -71.363 |
| AGP-01 | Amacayacu: Agua Pudre E | WA | 1 | 1992.210 | 2006.110 | -3.722 | -70.305 |
| AGP-02 | Amacayacu: Agua Pudre U | WA | 1 | 1991.871 | 2006.121 | -3.719 | -70.305 |
| ALF-01 | Alta Floresta plot 1 | SA | 1 | 2002.417 | 2011.450 | -9.598 | -55.937 |
| ALF-02 | Alta Floresta plot 2 | SA | 1 | 2008.405 | 2011.441 | -9.578 | -55.918 |
| ALM-01 | Altos de Maizal | WA | 2 | 1994.701 | 2008.669 | -11.800 | -71.467 |
| ALP-01 | Allpahuayo A | WA | 1 | 1991.871 | 2011.172 | -3.949 | -73.434 |
| ALP-02 | Allpahuayo B | WA | 1 | 1990.871 | 2011.181 | -3.953 | -73.437 |
| ALP-30 | Allpahuayo C | WA | 1 | 2001.260 | 2011.175 | -3.954 | -73.426 |
| ALP-40 | Allpahuayo D | WA | 1 | 2006.921 | 2011.189 | -3.941 | -73.439 |
| AMA-02 | Amargal 2 | Choco | 1 | 2006.414 | 2010.960 | 5.581 | -77.501 |
| ARA-01 | Arawete-Xingu | EA | 1 | 1986.078 | 1986.078 | -4.817 | -52.517 |
| ASR-01 | Asurini-Xingu | EA | 1 | 1986.454 | 1986.454 | -4.756 | -52.600 |
| BAC-01 | BACA-51162, Caparo | NSA | 0.25 | 1991.279 | 2009.140 | 7.417 | -70.833 |
| BAC-02 | BACA-51284, Caparo | NSA | 0.25 | 1991.860 | 2009.129 | 7.417 | -70.833 |
| BAC-03 | BACA-51294, Caparo | NSA | 0.25 | 1991.279 | 2009.129 | 7.417 | -70.833 |
| BAC-04 | BACA-51367, Caparo | NSA | 0.25 | 1991.860 | 2009.140 | 7.417 | -70.833 |
| BAC-05 | BACA-52301, Caparo | NSA | 0.25 | 2001.260 | 2009.140 | 7.417 | -70.833 |
| BAC-06 | BACA-52312, Caparo | NSA | 0.25 | 1996.309 | 2009.140 | 7.417 | -70.833 |
| BDF-01 | BDFFP, 2303 Dimona 5-6 | EA | 2 | 1985.285 | 2007.534 | -2.341 | -60.097 |
| BDF-03 | BDFFP, 1101 Gaviao | EA | 1 | 1981.129 | 2009.329 | -2.424 | -59.855 |
| BDF-04 | BDFFP, 1102 Gaviao | EA | 1 | 1981.129 | 2009.581 | -2.427 | -59.853 |
| BDF-05 | BDFFP, 1103 Gaviao | EA | 1 | 1981.200 | 2009.329 | -2.426 | -59.851 |
| BDF-06 | BDFFP, 1201 Gaviao | EA | 3 | 1981.501 | 2009.329 | -2.415 | -59.857 |
| BDF-07 | BDFFP, 1105 Gaviao | EA | 1 | 1981.619 | 2009.496 | -2.400 | -59.900 |
| BDF-08 | BDFFP, 1109 Gaviao | EA | 1 | 1981.619 | 2009.496 | -2.400 | -59.900 |
| BDF-09 | BDFFP, 1113 Florestal | EA | 1 | 1987.041 | 2007.038 | -2.397 | -59.846 |
| BDF-10 | BDFFP, 1301 Florestal 1 | EA | 2 | 1983.452 | 2007.038 | -2.389 | -59.855 |
| BDF-11 | BDFFP, 1301 Florestal 2 | EA | 3 | 1983.452 | 2007.038 | -2.385 | -59.850 |
| BDF-12 | BDFFP, 1301 Florestal 3 | EA | 2 | 1983.452 | 2007.038 | -2.392 | -59.853 |
| BDF-13 | BDFFP, 3402 Cabo Frio | EA | 9 | 1985.871 | 2009.871 | -2.399 | -59.914 |
| BDF-14 | BDFFP, 3304 Porto Alegre | EA | 1 | 1984.199 | 2009.200 | -2.364 | -59.974 |
| BEE-01 | BEEM plot 1 | SA | 1 | 2002.030 | 2010.047 | -16.533 | -64.583 |
| BEE-05 | BEEM plot 5 | SA | 1 | 2002.660 | 2010.047 | -16.533 | -64.583 |
| BNT-01 | Bionte 1 | EA | 1 | 1986.542 | 2010.534 | -2.643 | -60.158 |
| BNT-02 | Bionte 2 | EA | 1 | 1986.542 | 2010.534 | -2.642 | -60.150 |
| BNT-04 | Bionte 4 | EA | 1 | 1986.542 | 2010.534 | -2.629 | -60.154 |
| BNT-05 | Bionte T4 B2 SB1 | EA | 1 | 1986.542 | 1993.542 | -2.633 | -60.167 |
| BNT-06 | Bionte T4 B1 SB3 | EA | 1 | 1986.542 | 1993.542 | -2.633 | -60.167 |
| Code | Name | Region | Area  ha | First census | Last census | Lat.  dec. | Long.  dec. |
| BNT-07 | Bionte T4 B4 SB4 | EA | 1 | 1986.542 | 1993.542 | -2.633 | -60.167 |
| BOG-01 | Bogi 1 | WA | 1 | 1996.261 | 2011.537 | -0.702 | -76.479 |
| BOG-02 | Bogi 2 | WA | 1 | 1996.261 | 2011.545 | -0.700 | -76.471 |
| CAI-05 | BACAI-05, El Caimital | NSA | 0.25 | 1963.129 | 2009.121 | 8.667 | -70.217 |
| CAI-06 | BACAI-06, El Caimital | NSA | 0.25 | 1963.129 | 2009.121 | 8.667 | -70.217 |
| CAX-02 | Caxiuana 2 | EA | 1 | 1995.501 | 2009.871 | -1.743 | -51.461 |
| CAX-06 | TORRE Caxiuana | EA | 1 | 2004.607 | 2009.871 | -1.720 | -51.458 |
| CHO-01 | Chore 1 | SA | 1 | 1996.541 | 2001.449 | -14.386 | -61.148 |
| CNG-01 | Cerro Neblina Gentry | WA | 1 | 1984.298 | 1984.298 | 0.833 | -66.167 |
| CPP-01 | Fazenda Santo Amaro 1 | EA | 1 | 1997.452 | 2000.454 | -1.844 | -47.104 |
| CPP-02 | Fazenda Santo Amaro 2 | EA | 1 | 2002.452 | 2002.452 | -1.844 | -47.104 |
| CRG-01 | Carajas, Rio Gelado | EA | 1 | 1985.496 | 1985.496 | -5.900 | -50.133 |
| CRP-01 | Cerro Pelao 1 | SA | 1 | 1994.085 | 2011.436 | -14.538 | -61.500 |
| CRP-02 | Cerro Pelao 2 | SA | 1 | 1994.290 | 2011.432 | -14.538 | -61.498 |
| CUZ-01 | Cuzco Amazonico, 1E | WA | 1 | 1989.389 | 2011.690 | -12.499 | -68.974 |
| CUZ-02 | Cuzco Amazonico, 1U | WA | 1 | 1989.400 | 2011.685 | -12.499 | -68.971 |
| CUZ-03 | Cuzco Amazonico, 2E | WA | 1 | 1989.419 | 2011.696 | -12.500 | -68.963 |
| CUZ-04 | Cuzco Amazonico, 2U | WA | 1 | 1989.441 | 2011.707 | -12.499 | -68.960 |
| DOI-01 | RESEX Chico Mendes: Seringal Dois Irmãos 1 | WA | 1 | 1991.329 | 2011.452 | -10.567 | -68.311 |
| DOI-02 | RESEX Chico Mendes: Seringal Dois Irmãos 2 | WA | 1 | 1999.501 | 2011.452 | -10.550 | -68.307 |
| ELD-01 | El Dorado, km 93, plotG1 | NSA | 0.25 | 1971.551 | 2009.079 | 6.102 | -61.403 |
| ELD-02 | El Dorado, km 93, plotG2 | NSA | 0.25 | 1971.551 | 2009.079 | 6.102 | -61.404 |
| ELD-03 | El Dorado, km 98, plotG3 | NSA | 0.25 | 1971.551 | 2009.079 | 6.083 | -61.405 |
| ELD-04 | El Dorado, km 98, plotG4 | NSA | 0.25 | 1971.551 | 2009.079 | 6.083 | -61.406 |
| FEC-01 | Fazenda Experimental Catuaba | WA | 1 | 2000.858 | 2011.452 | -10.067 | -67.617 |
| FLO-01 | Fazenda Floresta, Ribeirão Cascalheira - Plot 1 | SA | 1 | 2008.356 | 2011.278 | -12.813 | -51.854 |
| FMH-01 | Forest reserve Mabura hill 01, Brown sand-Greenhart plot | EA (GS) | 1 | 1993.786 | 2010.169 | 5.173 | -58.693 |
| FMH-02 | Forest reserve Mabura hill 02, Brown sand-Greenhart plot | EA (GS) | 1 | 1993.767 | 2010.177 | 5.174 | -58.694 |
| FMH-03 | Forest reserve Mabura hill 03, White Sand plot | EA (GS) | 1 | 2005.619 | 2010.160 | 5.179 | -58.704 |
| INF-01 | Infierno | WA | 1.3 | 1988.790 | 1995.959 | -12.733 | -69.700 |
| JAC-01 | Jacaranda, norte-sul, plots 1-5 | EA | 5 | 1996.500 | 2010.534 | -2.606 | -60.207 |
| JAC-02 | Jacaranda, leste-oeste, plots 6-10 | EA | 5 | 1996.500 | 2010.534 | -2.615 | -60.196 |
| JAM-01 | Rio Jurua_AM | EA | 4 | 1990.496 | 1990.496 | -4.667 | -66.172 |
| JAS-02 | Jatun Sacha 2 | WA | 1 | 1987.619 | 2011.503 | -1.070 | -77.616 |
| JAS-04 | Jatun Sacha 4 | WA | 1 | 1994.414 | 2011.510 | -1.073 | -77.612 |
| JEN-11 | Jenaro Herrera A | WA | 1 | 2005.170 | 2011.285 | -4.878 | -73.630 |
| Code | Name | Region | Area  ha | First census | Last census | Lat.  dec. | Long.  dec. |
| JRI-01 | Jari 1 | EA | 1 | 1985.501 | 1996.500 | -0.894 | -52.190 |
| LAS-02 | Jacaratia Los Amigos | WA | 1 | 2004.372 | 2008.600 | -12.572 | -70.092 |
| LFA-01 | Jacazada_3-ALFA | EA | 1 | 1987.496 | 1987.496 | -5.850 | -50.480 |
| LFB-01 | Los Fierros Bosque I | SA | 1 | 1993.619 | 2011.396 | -14.579 | -60.831 |
| LFB-02 | Los Fierros Bosque II | SA | 1 | 1993.641 | 2011.402 | -14.577 | -60.832 |
| LOR-01 | Amacayacu: Lorena E | WA | 1 | 1992.501 | 2006.151 | -3.056 | -69.991 |
| LOR-02 | Amacayacu: Lorena U subplot 1-13 | WA | 0.52 | 1992.503 | 2006.151 | -3.057 | -69.993 |
| LOR-03 | Amacayacu: Lorena U subplot 14-25 | WA | 0.48 | 1992.506 | 2006.151 | -3.057 | -69.993 |
| LSL-01 | Las Londras, plot 1 | SA | 1 | 1996.500 | 2009.540 | -14.404 | -61.141 |
| LSL-02 | Las Londras, plot 2 | SA | 1 | 1996.500 | 2009.545 | -14.404 | -61.139 |
| MBT-01 | Mabet plot 01 | WA | 1 | 1999.690 | 2011.741 | -10.067 | -65.889 |
| MBT-02 | Mabet plot 02 | WA | 1 | 1999.762 | 2011.745 | -10.051 | -65.889 |
| MBT-04 | Mabet Plot 4 | WA | 1 | 2003.433 | 2011.761 | -10.306 | -65.555 |
| MBT-05 | Mabet plot 5 | WA | 1 | 2003.468 | 2011.758 | -10.033 | -65.628 |
| MBT-06 | Mabet plot 6 | WA | 1 | 2003.477 | 2011.755 | -10.037 | -65.641 |
| MBT-07 | Mabet plot 7 | WA | 1 | 2003.866 | 2011.752 | -9.912 | -65.739 |
| MBT-08 | Mabet plot 8 | WA | 1 | 2003.877 | 2011.750 | -9.941 | -65.751 |
| MIN-01 | Rio das Minas, Parque Nacional da Serra do Divisor | WA | 1 | 1996.497 | 2011.440 | -8.565 | -72.883 |
| MNU-01 | Manu, alluvial Cocha Cashu Trail 3, M1 | WA | 1 | 1974.710 | 2000.710 | -11.887 | -71.406 |
| MNU-03 | Manu, terra firme terrace, M3 | WA | 2 | 1991.701 | 2007.501 | -11.900 | -71.400 |
| MNU-05 | Manu, alluvial Cocha Cashu Trail 12 | WA | 2 | 1989.781 | 2008.639 | -11.879 | -71.408 |
| MNU-06 | Manu, alluvial Cocha Cashu Trail 2 & 31 | WA | 2.25 | 1989.816 | 2008.631 | -11.886 | -71.397 |
| MNU-08 | Cocha Salvador Manu, mature floodplain | WA | 2 | 1991.770 | 2007.501 | -11.995 | -71.235 |
| MRB-01 | Marabá: UA1 | EA | 2 | 1988.369 | 1995.959 | -5.733 | -49.050 |
| MRB-02 | Marabá: UA2 | EA | 2 | 1988.459 | 1995.959 | -5.717 | -49.033 |
| MRB-03 | Marabá: UA3 | EA | 2 | 1988.459 | 1995.959 | -5.700 | -49.000 |
| MSH-01 | Mishana | WA | 1 | 1983.019 | 1990.681 | -3.783 | -73.500 |
| MTH-01 | Marechal Thaumaturgo | WA | 1 | 1996.415 | 2011.443 | -8.884 | -72.790 |
| NOU-01 | Nouragues GP 10L | EA (GS) | 1 | 1993.197 | 2008.852 | 4.089 | -52.674 |
| NOU-02 | Nouragues GP 11L | EA (GS) | 1 | 1994.874 | 2008.852 | 4.088 | -52.675 |
| NOU-03 | Nouragues GP 12L | EA (GS) | 1 | 1993.370 | 2008.880 | 4.088 | -52.675 |
| NOU-04 | Nouragues GP 13L | EA (GS) | 1 | 1992.246 | 2008.869 | 4.087 | -52.676 |
| NOU-05 | Nouragues GP 14L | EA (GS) | 1 | 1992.746 | 2008.853 | 4.086 | -52.676 |
| NOU-06 | Nouragues GP 15L | EA (GS) | 1 | 1992.831 | 2008.869 | 4.085 | -52.677 |
| NOU-07 | Nouragues GP 16L | EA (GS) | 1 | 1993.159 | 2008.880 | 4.084 | -52.677 |
| NOU-08 | Nouragues GP 17L | EA (GS) | 1 | 1993.175 | 2008.891 | 4.084 | -52.678 |
| NOU-09 | Nouragues GP 18L | EA (GS) | 1 | 1993.438 | 2008.889 | 4.083 | -52.678 |
| NOU-10 | Nouragues GP 19L | EA (GS) | 1 | 1994.433 | 2008.899 | 4.086 | -52.678 |
| Code | Name | Region | Area  ha | First census | Last census | Lat.  dec. | Long.  dec. |
| NOU-12 | Nouragues PP 21H | EA (GS) | 1 | 1992.577 | 2008.910 | 4.082 | -52.682 |
| NOU-13 | Nouragues PP 22H | EA (GS) | 1 | 1992.593 | 2008.910 | 4.081 | -52.683 |
| NOU-15 | Nouragues PP 21G | EA (GS) | 1 | 1992.571 | 2008.929 | 4.083 | -52.683 |
| NOU-16 | Nouragues PP 22G | EA (GS) | 1 | 1992.596 | 2008.929 | 4.082 | -52.684 |
| NOU-17 | Nouragues PP 20F | EA (GS) | 1 | 1992.522 | 2008.929 | 4.084 | -52.683 |
| NOU-18 | Nouragues PP 21F | EA (GS) | 1 | 1992.538 | 2008.940 | 4.083 | -52.684 |
| NOU-19 | Nouragues PP 22F | EA (GS) | 1 | 1992.612 | 2008.940 | 4.082 | -52.684 |
| NOU-20 | Nouragues PP 20E | EA (GS) | 1 | 1992.642 | 2008.921 | 4.084 | -52.684 |
| NOU-21 | Nouragues PP 21E | EA (GS) | 1 | 1992.631 | 2008.937 | 4.084 | -52.685 |
| NOU-22 | Nouragues PP 22E | EA (GS) | 1 | 1992.626 | 2008.945 | 4.083 | -52.667 |
| NXV-04 | Cerradão do Ben Hur, Parque Municipal do Bacaba (UNEMAT) | SA | 0.5 | 2002.032 | 2010.034 | -14.700 | -52.351 |
| ODE-01 | O Deserto | EA | 3 | 1980.910 | 1980.910 | -3.483 | -51.667 |
| ODE-02 | O Deserto 2 | EA | 0.5 | 1980.910 | 1980.910 | -3.483 | -51.667 |
| PAR-20 | Guyaflux plot 1 | EA (GS) | 0.49 | 2004.404 | 2010.296 | 5.279 | -52.924 |
| PAR-21 | Guyaflux plot 2 | EA (GS) | 0.49 | 2004.495 | 2010.299 | 5.279 | -52.924 |
| PAR-22 | Guyaflux plot 3 | EA (GS) | 0.49 | 2004.481 | 2010.315 | 5.278 | -52.920 |
| PAR-23 | Guyaflux Plot 4 | EA (GS) | 0.49 | 2004.432 | 2010.304 | 5.280 | -52.922 |
| PAR-24 | Guyaflux plot 5 | EA (GS) | 0.49 | 2004.434 | 2010.307 | 5.281 | -52.920 |
| PAR-25 | Guyaflux plot 6 | EA (GS) | 0.49 | 2004.434 | 2010.307 | 5.281 | -52.919 |
| PAR-27 | Guyaflux plot 8 | EA (GS) | 0.49 | 2004.432 | 2010.304 | 5.280 | -52.922 |
| PAR-28 | Guyaflux plot 9 | EA (GS) | 0.49 | 2004.421 | 2010.296 | 5.278 | -52.924 |
| PAR-29 | Guyaflux plot 10 | EA (GS) | 0.49 | 2004.473 | 2010.315 | 5.278 | -52.922 |
| PAY-01 | Payamino | WA | 1 | 1987.786 | 1987.786 | -0.450 | -77.033 |
| PEA-02 | Impuca2 (Parque Estadual do Araguaia- PEA) | SA | 1 | 2007.674 | 2010.581 | -12.320 | -50.738 |
| PIB-06 | Pibiri 06 | EA | 1 | 1993.665 | 2010.207 | 5.013 | -58.625 |
| PIB-12 | Pibiri 12 | EA | 1 | 1993.665 | 2010.184 | 5.026 | -58.604 |
| PNY-04 | Paujil Venado Bosque Humedo Tropical | WA | 1 | 2007.547 | 2011.822 | -10.341 | -75.253 |
| PNY-05 | Paujil-Venado Bosque Humedo Tropical | WA | 1 | 2008.194 | 2011.811 | -10.350 | -75.250 |
| PNY-06 | Paujil-Venado Bosque Humedo Tropical | WA | 1 | 2008.206 | 2011.814 | -10.356 | -75.254 |
| PNY-07 | Paujil-Venado Bosque Humedo Tropical | WA | 1 | 2008.219 | 2011.816 | -10.347 | -75.259 |
| POR-01 | RESEX Chico Mendes: Seringal Porongaba 1 | WA | 1 | 1991.419 | 2011.443 | -10.818 | -68.776 |
| POR-02 | RESEX Chico Mendes: Seringal Porongaba 2 | WA | 1 | 1991.430 | 2011.452 | -10.800 | -68.773 |
| PPB-02 | Peixe-Boi Parcela 02 | EA | 1 | 1991.501 | 1999.501 | -1.183 | -47.317 |
| PPB-03 | Peixe-Boi Parcela 03 | EA | 1 | 1991.501 | 1999.501 | -1.183 | -47.317 |
| PTB-01 | Porto Trombetas 1 | EA | 1 | 1997.641 | 2007.121 | -1.166 | -56.414 |
| PTB-02 | Porto Trombetas 2 | EA | 1 | 1997.641 | 2007.121 | -1.478 | -56.386 |
| RBR-01 | Rondonia BR-364 | SA | 1 | 1986.496 | 1986.496 | -11.000 | -61.950 |
| RET-05 | Reserva El Tigre 05 | SA | 1 | 1995.218 | 2011.604 | -10.971 | -65.715 |
| Code | Name | Region | Area  ha | First census | Last census | Lat.  dec. | Long.  dec. |
| RET-06 | Reserva El Tigre 06 | SA | 1 | 1995.222 | 2011.610 | -10.971 | -65.715 |
| RET-08 | Reserva El Tigre 08 | SA | 1 | 1995.176 | 2011.615 | -10.971 | -65.715 |
| RET-09 | Reserva El Tigre 09 | SA | 1 | 1995.166 | 2011.619 | -10.971 | -65.715 |
| RIA-01 | Reserva Indigena Alto Turiacu | EA | 4 | 1991.496 | 1991.496 | -2.900 | -46.150 |
| RIO-01 | Rio Grande, plotDA1 | NSA | 0.25 | 1971.570 | 2009.079 | 8.114 | -61.692 |
| RIO-02 | Rio Grande, plotDA2 | NSA | 0.25 | 1971.570 | 2009.079 | 8.114 | -61.692 |
| RPA-01 | Rio Pariamanu | WA | 1 | 1998.915 | 1998.915 | -12.392 | -69.360 |
| RPI-01 | Rio Piedras | WA | 1 | 1995.581 | 1995.581 | -12.360 | -69.234 |
| RST-01 | Base da Restauração - Reserva Extrativista do Alto Juruá | WA | 1 | 1995.414 | 2011.435 | -9.039 | -72.267 |
| RTH-01 | Rio Tahuamanu | WA | 1 | 1996.497 | 1996.497 | -11.370 | -69.660 |
| SAA-01 | Floresta Santana do Araguaia-Pará | EA | 1 | 2011.414 | 2011.414 | -9.794 | -51.878 |
| SCR-04 | San Carlos de Rio Negro, MAB site, Tall Caatinga, plot A | EA | 1 | 1975.540 | 2006.200 | 1.927 | -67.036 |
| SCR-05 | San Carlos de Rio Negro, MAB site, Yevaro, plot B | EA | 1 | 1975.619 | 2006.249 | 1.930 | -67.038 |
| SCT-01 | Sacta plot 1 | SA | 1 | 2001.849 | 2011.685 | -17.087 | -64.768 |
| SCT-06 | Sacta Plot 6 | SA | 1 | 2002.619 | 2011.690 | -17.090 | -64.768 |
| SHI-01 | Shiripuno | WA | 1 | 1994.789 | 1994.789 | -1.019 | -76.976 |
| SUC-01 | Sucusari A | WA | 1 | 1992.113 | 2011.246 | -3.252 | -72.907 |
| SUC-02 | Sucusari B | WA | 1 | 1992.123 | 2011.246 | -3.253 | -72.903 |
| SUC-03 | Sucusari C | WA | 1 | 2001.079 | 2011.236 | -3.247 | -72.925 |
| SUC-04 | Sucusari D | WA | 1 | 2001.159 | 2011.266 | -3.251 | -72.892 |
| SUC-05 | Sucusari E | WA | 1 | 2001.121 | 2011.259 | -3.257 | -72.894 |
| TAM-01 | Tambopata plot zero | WA | 1 | 1983.781 | 2011.658 | -12.844 | -69.288 |
| TAM-02 | Tambopata plot one | WA | 1 | 1979.871 | 2011.663 | -12.835 | -69.286 |
| TAM-03 | Tambopata plot two swamp | WA | 0.58 | 1983.680 | 2011.644 | -12.837 | -69.278 |
| TAM-04 | Tambopata plot two swamp edge clay | WA | 0.42 | 1983.680 | 2011.647 | -12.837 | -69.278 |
| TAM-05 | Tambopata plot three | WA | 1 | 1983.688 | 2011.652 | -12.830 | -69.271 |
| TAM-06 | Tambopata plot four | WA | 1 | 1983.710 | 2011.673 | -12.838 | -69.296 |
| TAM-07 | Tambopata plot six | WA | 1 | 1983.751 | 2011.622 | -12.826 | -69.261 |
| TAM-08 | Tambopata plot seven | WA | 1 | 2001.529 | 2011.214 | -12.826 | -69.269 |
| TAM-09 | Tambopata plot eight | WA | 1 | 2010.687 | 2011.647 | -12.831 | -69.285 |
| TAN-04 | Fazenda Tanguro, plot 4 | SA | 1 | 2008.370 | 2011.284 | -12.921 | -52.373 |
| TAP-50 | Tapajos, RP014, 1 | EA | 0.25 | 1983.501 | 1995.501 | -3.309 | -54.940 |
| TAP-51 | Tapajos, RP014, 2 | EA | 0.25 | 1983.501 | 1995.501 | -3.309 | -54.940 |
| TAP-52 | Tapajos, RP014, 3 | EA | 0.25 | 1983.501 | 1995.501 | -3.309 | -54.940 |
| TAP-53 | Tapajos, RP014, 4 | EA | 0.25 | 1983.501 | 1995.501 | -3.309 | -54.940 |
| TAP-54 | Tapajos, RP014, 5 | EA | 0.25 | 1983.501 | 1995.501 | -3.310 | -54.945 |
| TAP-55 | Tapajos, RP014, 6 | EA | 0.25 | 1983.501 | 1995.501 | -3.310 | -54.945 |
| TAP-56 | Tapajos, RP014, 7 | EA | 0.25 | 1983.501 | 1995.501 | -3.310 | -54.945 |
| Code | Name | Region | Area  ha | First census | Last census | Lat.  dec. | Long.  dec. |
| TAP-58 | Tapajos, RP014, 9 | EA | 0.25 | 1983.501 | 1995.501 | -3.310 | -54.940 |
| TAP-59 | Tapajos, RP014, 10 | EA | 0.25 | 1983.501 | 1995.501 | -3.310 | -54.940 |
| TAP-60 | Tapajos, RP014, 11 | EA | 0.25 | 1983.501 | 1995.501 | -3.310 | -54.940 |
| TAP-61 | Tapajos, RP014, 12 | EA | 0.25 | 1983.501 | 1995.501 | -3.310 | -54.940 |
| TEC-01 | TEAM Caxiuana plot 1 | EA | 1 | 2002.871 | 2006.901 | -1.707 | -51.459 |
| TEC-02 | TEAM Caxiuana plot 2 | EA | 1 | 2003.189 | 2006.871 | -1.736 | -51.488 |
| TEC-03 | TEAM Caxiuana plot 3 | EA | 1 | 2003.219 | 2006.901 | -1.734 | -51.510 |
| TEC-04 | TEAM Caxiuana plot 4 | EA | 1 | 2003.310 | 2006.879 | -1.754 | -51.522 |
| TEC-05 | TEAM Caxiuana plot 5 | EA | 1 | 2003.476 | 2006.871 | -1.782 | -51.592 |
| TEC-06 | TEAM Caxiuana plot 6 | EA | 1 | 2003.329 | 2006.871 | -1.725 | -51.430 |
| TEM-03 | TEAM Manaus plot 3 | EA | 1 | 2003.933 | 2010.725 | -2.407 | -59.902 |
| TEM-04 | TEAM Manaus plot 4 | EA | 1 | 2003.889 | 2010.666 | -2.431 | -59.794 |
| TEM-05 | TEAM Manaus plot 5 | EA | 1 | 2004.027 | 2010.740 | -2.619 | -60.210 |
| TEM-06 | TEAM Manaus plot 6 | EA | 1 | 2004.175 | 2005.713 | -2.598 | -60.109 |
| TIP-01 | Tiputini 1 | WA | 1 | 1997.873 | 2011.554 | -0.661 | -76.404 |
| TIP-02 | Tiputini 2 | WA | 1 | 1997.641 | 2011.567 | -0.634 | -76.145 |
| TIP-03 | Tiputini 3 | WA | 1 | 1998.184 | 2011.574 | -0.639 | -76.154 |
| TMP-01 | Tambopata Pacal | WA | 2.25 | 1995.496 | 1995.496 | -13.135 | -69.568 |
| VCR-01 | Fazenda Vera Cruz plot 1 monodominant forest | SA | 0.6 | 1996.500 | 2011.293 | -14.831 | -52.160 |
| VCR-02 | Fazenda Vera Cruz mixed forest adjacent to monodominant forest | SA | 0.6 | 2003.585 | 2008.836 | -14.832 | -52.169 |
| YAN-01 | Yanamono A | WA | 1 | 1983.441 | 2011.218 | -3.440 | -72.846 |
| YAN-02 | Yanamono B | WA | 1 | 2001.129 | 2011.226 | -3.434 | -72.844 |
| ZAR-02 | Zafire Rebalse | WA | 1 | 2004.899 | 2009.761 | -4.002 | -69.904 |
| ZAR-03 | Zafire Terra Firme | WA | 1 | 2004.899 | 2009.761 | -3.995 | -69.900 |
| ZAR-04 | Zafire Altura | WA | 1.04 | 2005.121 | 2009.761 | -3.985 | -69.906 |
