# Supplementary material for: Fast demographic traits promote high diversification rates of Amazonian trees
Source: Ecol Lett. 2014 Mar 3;17(5):527–36. doi: 10.1111/ele.12252 (PMC4285998; doi:10.1111/ele.12252)
Supplement: Supplementary file 11 — supplementary [file ele0017-0527-SD11.docx]

**Table S3.** Species richness from (from Pennington *et al.* 2004; The Plant List 2010) and average turnover times calculated from forest dynamics data for 150 genera with >100 stems in 187 multiple census plots.

| Family | Genus | Turnover time | Species richness |
| --- | --- | --- | --- |
|  |  | years | no. |
| Anacardiaceae | *Anacardium* | 151 | 10 |
| Anacardiaceae | *Astronium* | 112 | 8 |
| Anacardiaceae | *Spondias* | 74 | 10 |
| Anacardiaceae | *Tapirira* | 44 | 10 |
| Anacardiaceae | *Thyrsodium* | 43 | 7 |
| Annonaceae | *Rollinia* | 38 | 45 |
| Annonaceae | *Ruizodendron* | 51 | 1 |
| Apocynaceae | *Aspidosperma* | 96 | 70 |
| Apocynaceae | *Geissospermum* | 499 | 5 |
| Araliaceae | *Dendropanax* | 52 | 75 |
| Bignoniaceae | *Jacaranda* | 59 | 50 |
| Bignoniaceae | *Tabebuia* | 96 | 100 |
| Bixaceae | *Bixa* | 36 | 5 |
| Boraginaceae | *Cordia* | 45 | 300 |
| Caricaceae | *Jacaratia* | 22 | 6 |
| Caryocaraceae | *Caryocar* | 227 | 16 |
| Celastraceae | *Maytenus* | 94 | 200 |
| Chrysobalanaceae | *Couepia* | 101 | 71 |
| Chrysobalanaceae | *Hirtella* | 78 | 107 |
| Chrysobalanaceae | *Licania* | 107 | 218 |
| Clusiaceae | *Caraipa* | 98 | 30 |
| Clusiaceae | *Garcinia* | 51 | 200 |
| Clusiaceae | *Symphonia* | 71 | 10 |
| Combretaceae | *Buchenavia* | 31 | 24 |
| Combretaceae | *Terminalia* | 97 | 200 |
| Dichapetalaceae | *Tapura* | 107 | 25 |
| Ebenaceae | *Diospyros* | 35 | 470 |
| Elaeocarpaceae | *Sloanea* | 74 | 120 |
| Euphorbiaceae | *Alchornea* | 32 | 50 |
| Euphorbiaceae | *Drypetes* | 78 | 200 |
| Euphorbiaceae | *Hieronima* | 60 | 15 |
| Euphorbiaceae | *Micrandra* | 147 | 7 |
| Euphorbiaceae | *Micrandropsis* | 410 | 1 |
| Euphorbiaceae | *Nealchornea* | 60 | 2 |
| Euphorbiaceae | *Sagotia* | 83 | 2 |
| Euphorbiaceae | *Sapium* | 66 | 21 |
| Fabaceae | *Abarema* | 135 | 40 |
| Fabaceae | *Clathrotropis* | 99 | 5 |
| Fabaceae | *Cynometra* | 93 | 70 |
| Fabaceae | *Mora* | 342 | 7 |
| Family | Genus | Turnover time | Species richness |
|  |  | years | no. |
| Goupiaceae | *Goupia* | 170 | 2 |
| Humiriaceae | *Endopleura* | 119 | 1 |
| Humiriaceae | *Sacoglottis* | 81 | 8 |
| Humiriaceae | *Vantanea* | 174 | 15 |
| Icacinaceae | *Calatola* | 71 | 7 |
| Icacinaceae | *Poraqueiba* | 94 | 3 |
| Lacistemataceae | *Lacistema* | 32 | 10 |
| Lauraceae | *Aniba* | 58 | 40 |
| Lauraceae | *Chlorocardium* | 420 | 2 |
| Lauraceae | *Endlicheria* | 38 | 60 |
| Lauraceae | *Licaria* | 79 | 40 |
| Lauraceae | *Mezilaurus* | 205 | 18 |
| Lauraceae | *Nectandra* | 30 | 110 |
| Lauraceae | *Ocotea* | 51 | 300 |
| Lauraceae | *Pleurothyrium* | 32 | 40 |
| Lecythidaceae | *Corythophora* | 210 | 4 |
| Lecythidaceae | *Couratari* | 208 | 20 |
| Lecythidaceae | *Eschweilera* | 140 | 90 |
| Lecythidaceae | *Gustavia* | 85 | 40 |
| Lecythidaceae | *Lecythis* | 180 | 26 |
| Linaceae | *Roucheria* | 35 | 7 |
| Malpighiaceae | *Byrsonima* | 39 | 150 |
| Malvaceae | *Apeiba* | 58 | 7 |
| Malvaceae | *Catostemma* | 102 | 15 |
| Malvaceae | *Eriotheca* | 132 | 20 |
| Malvaceae | *Lueheopsis* | 138 | 7 |
| Malvaceae | *Matisia* | 89 | 25 |
| Malvaceae | *Pachira* | 56 | 50 |
| Malvaceae | *Quararibea* | 114 | 30 |
| Malvaceae | *Scleronema* | 124 | 4 |
| Malvaceae | *Sterculia* | 92 | 200 |
| Melastomataceae | *Mouriri* | 99 | 80 |
| Meliaceae | *Trichilia* | 45 | 96 |
| Myristicaceae | *Osteophloeum* | 140 | 2 |
| Myrtaceae | *Myrcia* | 48 | 450 |
| Myrtaceae | *Psidium* | 68 | 70 |
| Olacaceae | *Heisteria* | 78 | 33 |
| Olacaceae | *Minquartia* | 130 | 1 |
| Polygonaceae | *Coccoloba* | 42 | 120 |
| Polygonaceae | *Triplaris* | 13 | 17 |
| Quiinaceae | *Quiina* | 49 | 35 |
| Rubiaceae | *Capirona* | 56 | 1 |
| Rubiaceae | *Chimarrhis* | 90 | 14 |
| Rutaceae | *Metrodorea* | 35 | 5 |
| Family | Genus | Turnover | Species |
|  |  | years | no. |
| Salicaceae | *Lunania* | 34 | 14 |
| Sapotaceae | *Chrysophyllum* | 123 | 80 |
| Sapotaceae | *Ecclinusa* | 151 | 11 |
| Sapotaceae | *Manilkara* | 180 | 30 |
| Sapotaceae | *Micropholis* | 67 | 40 |
| Sapotaceae | *Pouteria* | 109 | 350 |
| Sapotaceae | *Pradosia* | 288 | 23 |
| Sapotaceae | *Sarcaulus* | 104 | 5 |
| Ulmaceae | *Ampelocera* | 51 | 9 |
| Ulmaceae | *Celtis* | 55 | 75 |
| Violaceae | *Leonia* | 77 | 5 |
| Vochysiaceae | *Erisma* | 153 | 16 |
| Vochysiaceae | *Qualea* | 133 | 60 |
